# Inhalable nanoparticle-based delivery systems for the treatment of pulmonary infections: *Status quo* and barrier-overcoming strategies

**DOI:** 10.1080/10717544.2025.2544683

**Published:** 2025-08-11

**Authors:** Yihong Gao, Wenhao Wang, Xiao Yue, Guanlin Wang, Kaiqing Zhang, Chuanbin Wu, Ziyu Zhao, Zhengwei Huang, Xuejuan Zhang

**Affiliations:** a College of Pharmacy, Jinan University, Guangzhou, PR China; b School of Pharmaceutical Sciences, Sun Yat-sen University, Guangzhou, PR China; c Jiangmen Wuyi Hospital of Traditional Chinese Medicine, Affiliated Jiangmen Traditional Chinese Medicine Hospital of Jinan University, Jiangmen, Guangdong Province, China

**Keywords:** Pulmonary infections, nanoparticle-based delivery system, physiological barrier, mucus barrier, biofilm barrier

## Abstract

Pulmonary infection is a serious public health challenge with high morbidity and mortality. The employment of antibiotics is the first-line treatment for pulmonary infections, while other novel anti-infection agents, such as antimicrobial peptides, have also been developed due to the emergence of drug resistance. Recently, inhalable nanoparticle-based delivery systems have garnered significant attention for the delivery of anti-infection agents, which possess great advantages like high lung accumulations and precise delivery performances. However, the respiratory physiological structure, mucus and biofilm have been considered as the barriers that nanoparticle drug delivery systems facing, which compromise the therapeutic effects. In this integrative review, recent advances in the inhalable nanoparticle-based delivery system were introduced. In addition, we focused on the biological characteristics of these barriers and discussed effective strategies to overcome the obstacles, including precise deposition in the lower respiratory tract infection site, effective penetration of mucus and breaking of the biofilm barrier. To sum up, this review aimed to deepen the understanding of the fate of anti-infective nanoformulations in pulmonary delivery and find effective strategies to address the barriers, thus providing new insights for the development of pulmonary delivery systems against pulmonary infections.

## GRAPHICAL ABSTRACT

**Figure:**
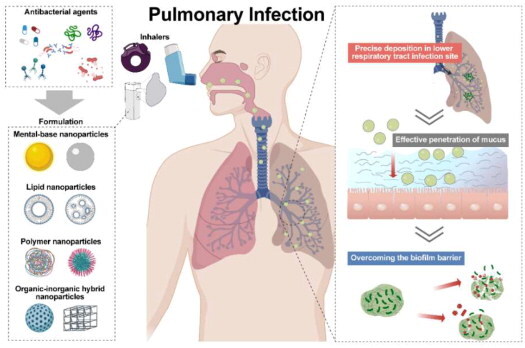

## 1. Introduction

Pulmonary infections have become a serious cause of death worldwide. As reported by the World Health Organization, pulmonary infections, which are also called lower respiratory infections, are the fourth cause of death worldwide with 2.5 million deaths in 2021 (World Health Organization 2024). Pathogens including bacteria, fungi, and viruses cause lung infections. Among these, bacteria account for the main causes of pulmonary infections, such as *Mycobacterium tuberculosis*, *Pseudomonas aeruginosa (PA), Staphylococcus aureus*, *Haemophilus influenzae*, and *Streptococcus Pneumoniae* (Roquilly et al. 2019; Deshpande and Zou 2020). Bacteria-induced infections of the lower respiratory cause pulmonary diseases such as tuberculosis and pneumonia, or can affect the course of related lung diseases such as cystic fibrosis (CF), chronic obstructive pulmonary disease (COPD), asthma, etc.

Antibiotic therapy is the main approach for treating bacterial infections in the clinic. Recently, emerging antibacterial agents, like antimicrobial peptides (AMPs), phages, and antibodies, have served as an effective strategy or supporting strategy for the treatment of infectious diseases (Li et al. 2023). These novel antibacterial agents shed light on the effective treatment of pulmonary infections. In addition, although these agents with antibacterial activity show great prospects, the suitable drug carrier is also of significance, as it can facilitate the efficient delivery to the lesion site. Therefore, selecting the optimal delivery carrier and employing the active agents are expected to promote the formulation to exert the ideal therapeutic effect. There are various kinds of carriers for drug delivery, like liposomes, microspheres, exosomes, and so on. With the development of nanotechnology, nanoparticles have shown great potential for drug delivery due to their small size, and the ability to co-deliver multiple drugs, among other advantages. The nanoparticles able to encapsulate the antibacterial agents have many advantages, including increasing the drug concentration, controlling the release behavior, and achieving targeted delivery (Zhang et al. 2010). Therefore, nanoparticles have great application prospects in delivering antibacterial agents for pulmonary infections.

A further issue lies in the administration route of the nanoparticles. Commonly, antibacterial agents are administered systemically via oral routes or intravenous administration (MacGregor and Graziani 1997). Pulmonary delivery, as a noninvasive method of drug delivery through the throat and bronchi, has many advantages (Zhang et al. 2017; Wang et al. 2022). Currently, pulmonary delivery is often achieved by inhalation dosage forms like dry powder inhalers (DPIs), nebulizers, metered dose inhalations (MDIs), and soft mist inhalations (SMIs) (Kole et al. 2023). Compared with the system treatment, the lung possesses limited drug-metabolic enzymes than the liver. Therefore, pulmonary administration can reduce the degradation by metabolic enzymes, thereby increasing the concentration of drugs (Loira-Pastoriza et al. 2014; Rubin et al. 2020). Moreover, the accumulation site is mainly limited to the lungs, which can also reduce the toxic effects of the drug on other tissues of the body and avoid irreversible damage. Based on this rationale, inhalable nanoparticles can be designed and developed, which will become an excellent choice for antibacterial agent delivery.

However, there are only several relevant products (like Arikayce^®^, TOBI^®^ Podhaler^™^, and Cayston^®^ (Li et al. 2023)) approved, which demonstrates the limited success in the clinical translation of inhalable antibacterial agent-accommodated nanoparticles. It must be pointed out that some barriers hamper the clinical translation. We state that the complex structure of the lower respiratory tract, mucus barrier, and biofilm barrier will reduce the concentration and therapeutic effect of antibacterial drugs, even for nanoparticle systems. It is necessary to deepen the understanding of these barriers that manipulate the fate of nanoparticles in pulmonary delivery and find effective strategies to surmount them to increase their therapeutic effects.

In this review, we first reviewed the antibacterial agents in the treatment of infectious diseases. Then, the progress of the inhalable nanoparticles drug delivery system for pulmonary infections was discussed. Furthermore, we described the barriers the system faced and outlined the corresponding strategies to overcome the above barriers. This review aimed to summarize the application of inhalable nanoformulations in the treatment of pulmonary infections. Providing effective strategies for addressing the barriers faced by pulmonary drug delivery would offer new insights for the development of pulmonary delivery systems against pulmonary infections.

## 2. The current therapy for pulmonary infections

At present, antibiotic therapy is the most conventional clinical therapeutic strategy, but this therapy faces challenges with the emergence of drug-resistant bacteria, due to the abuse of antibiotics. To date, a pipeline of new/non-conventional antibacterial therapeutics has emerged for the treatment of bacterial infection. These emerging antibacterial agents are mostly biologicals, including AMPs, phages, and antibodies. They are designed to selectively interact with a specific target and have been explored as alternatives or adjuvants for the treatment of bacterial infections. These novel candidates show great promise against antibiotic-resistant bacterial infections.

### 2.1. Antibiotics

The clinical therapy of pulmonary infections is mainly focused on antibiotics, including aminoglycosides, quinolones, *β*-lactam, sulfonamides, tetracyclines, and macrolides. For example, Tobramycin, amikacin lysine, and levofloxacin inhalation solutions have been approved in the European Union and Canada for the treatment of patients with chronic pulmonary infections with *PA* (Schwarz et al. 2021).

However, the overuse of antibiotics has allowed the rapid spread of drug-resistant bacteria, and antimicrobial resistance (AMR) bacterial infections have become a global health crisis. The use of antibiotics cannot effectively eliminate the infected bacteria, leading to the continuous deterioration of the disease, which can mainly be attributed to bacterial resistance and biofilm formation (de la Fuente-Nunez et al. 2023). Bacteria can develop resistance by reducing intracellular drug accumulation (reducing permeability and antibiotic efflux), altering bacterial antibiotic targets, destroying the drugs themselves, and bypassing entire metabolic pathways (Darby et al. 2023). Moreover, bacterial biofilms are naturally resistant and impermeable to antibiotics, and they are difficult to eradicate, making the bacteria exist for a long time, infectious diseases are easy to relapse, which will be further discussed in subsequent sections. Therefore, antibiotic monotherapy cannot completely eradicate bacteria, and multi-target combination therapy may stand out significantly in combating drug-resistant bacteria.

### 2.2. AMPs

Recent studies have illustrated that AMPs, ubiquitous in all forms of life as part of innate immune defenses, show good potential in anti-infection therapy (Zasloff 2002). AMPs are positively charged small-molecule compounds, usually composed of 10–50 amino acid residues, and can exert antibacterial activity by directly targeting pathogens through membrane-permeability properties (Zhang and Gallo 2016). Compared with traditional antibiotics, AMPs have outstanding advantages, they inhibit or eliminate bacteria through a membrane‐active mechanism rather than site‐specific binding or interference with bacterial metabolism (Mhlongo et al. 2023). Specifically, AMPs bind to the bacterial membrane through electrostatic forces and neutralize the charge of the bacterial membrane, thus destroying the integrity of the cell membrane and causing bacterial decomposition. This nonspecific mechanism endows AMPs with broad antibacterial activity, and AMPs have emerged as promising alternatives for treating pulmonary infections. Besides, AMPs can also diffuse through the plasma membrane of cells and accumulate in cells, thus blocking DNA replication and destroying RNA and protein synthesis (Rowe-Magnus et al. 2019).

An increasing number of anti-bacterial active AMPs were found to combat the infectious diseases. Among them, the AMP Esc(1–21) (Mangoni et al. 2024), AMP AA139, and SET-M33 (van der Weide et al. 2019) were found to be potent in anti-pulmonary infections. Until now, more than 3,000 AMPs have been reported and characterized, most of them are in their natural state and not suitable for human administration (Moretta et al. 2020). Seven AMPs molecules have been approved by the FDA for the treatment of bacterial infection: colistin, gramicidin, daptomycin, vancomycin, oritavancin, dalbavancin, and telavancin (Patrulea et al. 2020). Some AMPs are in clinical stage. Lactoferrin are endogenous antimicrobial proteins secreted by airway. ALX-009, composed of lactoferrin and hypothiocyanite, is an orphan drug developed by Alaxi. It was tested in a clinical trial to treat pulmonary disease and is given by inhalation (phase 1, NCT02598999) (further information can be found at http://clinicaltrials.gov).

However, AMPs therapeutics face the following challenges (Wang et al. 2022). First of all, the safety of AMPs is the top priority, since they are foreign substances with positive charge. AMPs can not only have a lethal effect on pathogens but can also target human cells in high concentrations. Vancomycin and colistin have been approved by the FDA, but some clinical studies have suggested that they may cause kidney or nerve damage in some patients or at high doses (Chen and Lu 2020). To overcome the nonselective effect, chemically modified AMPs, including glycosylation, lipidation, grafting, and cyclization, possess a selective interaction with bacteria and can reduce the toxicity to mammalian cells. Although the side effects of modified compounds such as olivanin and dabavancin are mild, some still remain some issues. The effectiveness and long-term therapeutic effect of modified AMPs against drug-resistant bacteria remain unclear (Smith et al. 2015; Syed and Scott 2015). Finally, as peptide drugs, the AMPs are likely to be degraded by the protease in lung, which could affect the activity to some extent. While AMPs exhibit many advantages over antibiotics in terms of reliability against drug resistance, it is significant to address some major limitations they hold that prevent the application in the treatment of pulmonary infections.

### 2.3. Phages therapy

Phages are viruses that infect and lyse specific bacteria. Different from common antibiotics, phage therapy has different antibacterial mechanisms, showing strong antibacterial properties against a variety of drug-resistant bacteria (Kalelkar et al. 2022).

After infecting bacteria, the phages will colonize the bacteria and multiply at the expense of the bacteria. First, natural or genetically modified phages will selectively target the bacteria, and the phage genome will integrate into the bacterial genome and then use the host machinery for replication and translation. At the same time, the expression of reporter proteins, toxins, lysins, and AMPs would damage the host cell. Further, the genome and the protein of the phage would assemble into progeny phage, which would trigger the cell lysis extensively. Besides, many phages produce depolymerases that degrade the biofilm matrix (Donlan 2009). Phages therapy can be used to combat common pathogenic bacteria causing pulmonary infections, including *Staphylococcus aureus* (Plumet et al. 2022), *PA* (Jennes et al. 2017) and *Acinetobacter baumannii* (Schooley et al. 2017), *etc*., and is a common therapeutic approach in parts of Eastern Europe (Harper and Enright 2011). Over the last few years, 20 cases of phage therapy for the treatment of pulmonary infections have been reported, mostly by expert centers in the US and Europe (Mitropoulou et al. 2022). A phage product candidate AP-PA02 developed by Armata Pharmaceuticals for P. aeruginosa infection in CF patients is currently in a phase 1b/2 clinic trial (NCT04596319) (further information can be found at http://clinicaltrials.gov). And the clinical trials shed light on the phage therapy in pulmonary infections, containing the clinical applications and key considerations.

However, there are concerns about the application of phage therapy. First, the specificity of the receptor for a single phage strain will determine its host range. High specificity may be a disadvantage, leading to an inability to kill all bacteria, especially in the case of polymicrobial biofilms, and designing phage mixtures with broad-spectrum activity can overcome the limitations of natural host specificity (Donlan 2009). The low specificity may have a killing effect on the commensal flora. The phages with the appropriate specificity can lyse certain bacteria, but it is a great challenge to find or design such phages. Therefore, the interaction between the pulmonary infection-related bacteria and the phages should be further investigated, which could make a contribution to finding or designing the specific phages. In addition, bacterial resistance to phages has been reported, assessing the susceptibility of bacteria to a particular phage is therefore crucial (Labrie et al. 2010). More important, the safety of phage preparations cannot be ignored. On the one hand, phages may encode virulence factors of several different organisms, including extracellular toxins or proteins required for host attachment, cell invasion, and intracellular survival. These genes may be integrated into the host genome during the process of parasitic bacteria, and the transfer of these genes should be avoided. On the other hand, the use of phages formulations may cause an immune response in the human body (Abedon et al. 2011).

In recent years, related technologies such as synthetic biology and genetic engineering have gradually overcome these limitations. For example, receptor-binding protein (RBP) engineering is utilized to expand and adapt the host range of phages, reporter phage is used for rapid screening of phage susceptibility characteristics gene-editing technology can precisely design specific genes or gene clusters in phage genomes. These technologies endow phages with additional therapeutic power, higher safety, and an adaptable host range (Meile et al. 2022), and the phages therapy has emerged as promising alternatives for treating pulmonary infections.

### 2.4. Antibodies

Antibodies have been widely used in the therapy and diagnosis of diseases, which is mainly attributed to their clear and highly specific targeted binding ability. Toxins and bacterial surface components are ideal sites targeting for antibodies. In the treatment of infectious diseases, monoclonal antibodies can exert antibacterial effects through a variety of mechanisms, like toxin neutralization, virulence factor inhibition, complement-mediated killing activity, and regulated phagocytosis (Wang et al. 2022). A number of mAbs have been shown to block infection in rodents models. With the development of global pandemic of severe acute respiratory syndrome coronavirus 2 (SARS-CoV-2) infection, two kinds of inhaled anti-SARS-CoV-2 antibodies were verified the ability to blunt the SARS-CoV-2 infection in macaques limits viral replication and lung pathology (Streblow et al. 2023). And three antimicrobial monoclonal antibodies have been approved by the FDA for clinical use, Raxibacumab (Abthrax^®^), Obiltoxaximab (Anthim^®^), and Bezlotoxumab (Zinplava^®^) (Motley et al. 2019), and they all exert the effect through toxin neutralization. There is no marketed antibody drug for pulmonary infections, but some related drugs have entered the clinical research stage. For example, a Phase III clinical study (CTR20231511) (further information can be found at http://www.chinadrugtrials.org.cn) is evaluating the efficacy and safety of MEDI3506 injection containing Tozorakimab, which was conducted by patients to treat the viral pulmonary infections requiring oxygen therapy.

There are also many candidates in preclinical or clinical trials. However, some candidate antibodies fail in clinical trials due to problems such as poor efficacy and stability. Antibodies only target specific antigens, which are unavailable to eliminate bacteria. Moreover, bacterial virulence factors are complex and change constantly in organisms, making single antibodies less effective (Zurawski and McLendon 2020). Despite the challenges, it may be possible to enhance the targeting-specificity of virulence factors via antibody mixtures and bis-specific or even poly-specific antibodies. Numerous studies have demonstrated that the combination of antibodies and antibiotics can effectively treat bacterial infections (Mariathasan and Tan 2017; Kirui et al. 2019; Kajihara et al. 2021). With the in-depth study of bacterial pathogenic mechanisms and the continuous development of antibody drugs, we believe the antibodies would play an increasingly important role in the treatment of antibiotic-resistant bacterial pulmonary infections, either alone or in combination with other therapeutics.

### 2.5. Bacteriocins

Many commensal bacteria can produce small antimicrobial molecules called bacteriocins that have the ability to eliminate specific pathogens. Bacteriocins have attracted increasing attention as potential microbiome editing tools, which are special antimicrobial peptides generated by ribosomes both in Gram-positive and Gram-negative bacteria (Daw and Falkiner 1996; Jong et al. 2011).

The antimicrobial mechanisms of bacteriocins are highly diverse and often cannot be fully elucidated due to the complexity of bacterial life activities (Simons et al. 2020). These mechanisms may revolve around the degradation of essential cellular components to the process of inhibiting specific molecular targets, such as the disassembly of enzymes required for the synthesis of cell walls, proteins or nucleic acids to bacterial membranes. Bacteriocins can be classified based on size or physicochemical properties. For example, the related protein stability and thermal stability or the expression of characteristic chemical moieties can be the basis for classification (Behrens et al. 2017). In addition, many bacteriocins are divided according to the taxon (bacterial family or genus) that produces them, such as the microcins (Yang et al. 2024), enterocins (Wu et al. 2022), and staphylococcins (de Freire Bastos et al. 2020), which are produced by *Enterobacteriaceae*, *Enterococci* and *Staphylococci*, respectively.

Many new bacteriocins show broad-spectrum antimicrobial activity such as *Methicillin-resistant Staphylococcus aureus (MRSA)*, and *PA* (Liu et al. 2020; Walsh et al. 2021), which are the common pathogenic bacteria in lung. The antibacterial efficacy of S-pyocins (Redero et al. 2020) and chimeric bacteriocin S5-PmnH (Paškevičius et al. 2022) has been demonstrated in vitro and acute lung infection models. However, the application of bacteriocins is limited by two aspects. First of all, bacteriocin is produced to promote or prevent the invasion of new bacterial strains into the microbial community, which is the adaptability characteristic of microorganisms to the environment. However, the interaction between bacteria is a delicate balance between antagonism and mutual benefit (Majeed et al. 2013). At present, the life activities of bacteria are not well clear, and the complexity of this process needs to be further studied. Second, bacteriocin production is often a transient property that can be spontaneously acquired by bacterial strains through horizontal gene transfer (HGT) of BGC and can be lost by the deletion of biosynthetic genes (Christenson and Gordon 2009). This implies that bacteriocin production is not a universal means of ensuring lineage success, but rather an adaptation to demands within specific microbial communities and spatial habitats, and its production is erratic (Heilbronner et al. 2021). Thus, only by having a full understanding of the various functions of bacteriocins can we understand how they shape the competitive fitness of producers and use specific bacteriocins for targeted microbiome interventions.

These novel candidates demonstrate promising application against bacterial infections. If they are prepared as nanoformulations, it is more conducive to the treatment of pulmonary infections.

## 3. Inhalable nanoparticle-based delivery system for pulmonary infections

In general, oral and/or intravenous administration is the commonly used administration route for different drugs clinically. However, treatment of pulmonary infections with above-administered drugs requires high doses to maintain therapeutic concentrations, because only a small fraction of the administered drugs can access the mucosal side of the lungs from the systemic circulation. In contrast, the inhalation can deliver the drug to the primary site of pulmonary infections (namely, the lung), while minimizing systemic exposure and side effects. There have been some approved inhaled antibiotics by FDA to target bacterial lung infections, like Arikayce^®^, TOBI^®^ Podhaler^™^, and Cayston^®^ (Li et al. 2023). There remain some issues in conventional pulmonary delivery. Inhalable drugs often are rapidly removed from the lung or inactivated by metabolic enzymes, thus shortening the residence time and reducing the therapeutic effect of drugs. Therefore, although inhalation has great potential in the treatment of pulmonary infections, and it is necessary to develop efficient formulations to address the above issue.

**Figure 1.**
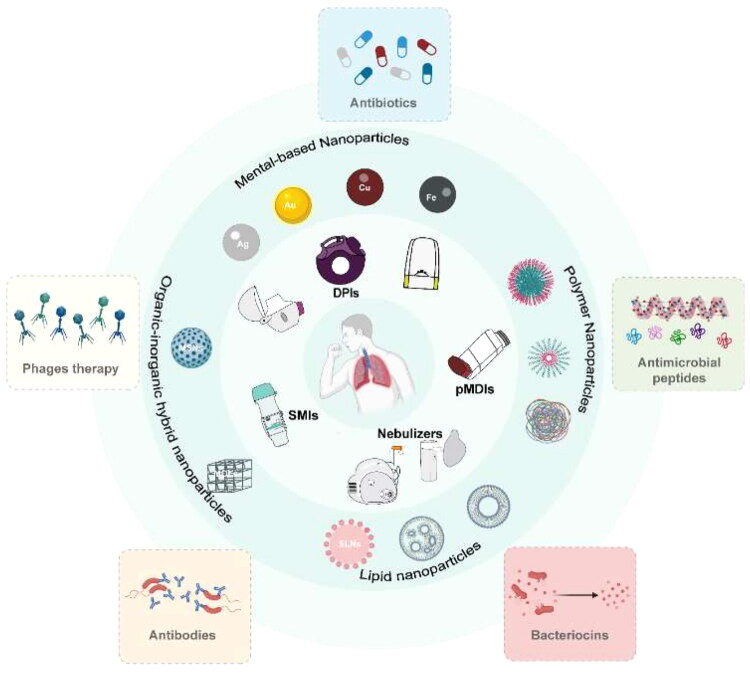
Schematic illustration of the inhalable nanoparticle-based delivery system for pulmonary infection. The DPIs, pMDIs, nebulizers and SMIs are the common inhalers. The nanoparticles, including mental-, polymer-, lipid-, and organic inorganic hybrid-nanoparticles are widely applied in the pulmonary delivery. Some efficient therapies are also mentioned. (originally created in adobe illustrator 2023).

For years, nanoparticle-based delivery technologies have emerged as attractive approaches to circumvent the limitations of conventional formulations administrated via oral, injectable or inhalable routes. Nanoparticles, as carriers, have shown great potential in increasing the accumulation of drugs, controlling and potentially sustaining drug release, decreasing the risk of adverse effects, and especially allowing combinatorial delivery of multiple antimicrobial agents together within one nanoparticle. Studies have shown that inhalable nanoformulations, like nanoparticles loading with ciprofloxacin (Al-Obaidi et al. 2018; Tran et al. 2019), polymyxin (Jasim et al. 2017), vancomycin (Abdelaziz et al. 2023) or other drugs, exhibit good delivery performances. Nowadays, many nanomaterials have been developed, including mental-, lipid-, polymer- and organic-inorganic hybrid nanomaterials. In the following content, we will review the research status of inhalable nanoformulations in the treatment of pulmonary infection (Figure 1).

### 3.1. Metal-based nanomaterials

Metal-based nanomaterials become promising antibacterial agents due to their tunable morphology, adjustable size, controlled drug release, and low risk of microbial resistance. They exert antibacterial effects through various mechanisms, including cell wall interaction, membrane penetration, reactive oxygen species (ROS) production, DNA damage, and inhibition of protein synthesis (Zheng et al. 2022). Silver-, gold-, iron-, and Cu-based nanoparticles showed significant antibacterial activity. These characteristics make metal-based nanoparticles promising candidates for developing novel antibacterial agents effective against pulmonary infections.

Silver ions can interact with sulfhydryl groups to interfere with the integrity of bacterial cell membranes, respiratory chain, enzyme activity and cell proliferation (Li et al. 2016). Silver-based nanoparticles (AgNPs) have been used as an excellent antibacterial agent against bacteria, especially multidrug-resistant bacteria. Besides, the combination of AgNPs and other materials improves antibacterial properties. For example, AgNPs can be combined with antibacterial agents such as organic compounds or antibiotics, showing synergistic effects against pathogen bacteria such as *Escherichia coli* (*E. coli*) and *S. aureus* (Bruna et al. 2021). The excellent pulmonary delivery performance of AgNPs has been demonstrated by many studies. Dultseva et al. (Valiulin et al. 2023) studied the aerosol inhalation delivery of composite particles composed of polyvinylpyrrolidone coated AgNPs and the survival rate of infected mice after aerosol exposure demonstrated the high antibacterial effect of AgNPs after inhalation. Due to excellent antibacterial and pulmonary delivery performance, the AgNPs show great potential in the pulmonary infections.

Au-based nanoparticles (AuNPs) could regulate intracellular uptake through surface charge or size adjustment, and inhibit intracellular ATP synthesis and tRNA binding, and the antibacterial mechanism of AuNPs is similar to others. In addition to inherent antibacterial properties, AuNPs also possess excellent photothermal properties, generating strong local heat under near-infrared radiation, resulting in high temperatures to kill bacteria. However, the precious metal nanoparticles, such as gold and platinum, are expensive. The combination with other material, like organic compounds or inorganic carbon can reduce costs and perform their functions.

Cu-based nanoparticles (CuNPs) can be in close contact with the biofilm and release free radicals to kill bacteria in a high oxidative environment. Then, these free radicals would oxidize and destroy the lipids of the microbial membrane, resulting in the leakage of cell contents and bacterial death (Din and Rehan 2017). This antibacterial mechanism called cuproptosis-like death has been studied in recent years (Luo et al. 2024; Xue et al. 2024). In addition, CuO nanoparticles, copper/carbon nanozymes (Xi et al. 2019), CuCo_2_O_4_ nanoflowers (Wang et al. 2024), and other materials would mimic peroxidase (POD) and oxidase (OXD) activities to exert catalytic activity and elevate ROS level, which subsequently induces bacteria to death. Due to the diversity of antibacterial mechanisms, CuNPs are widely used in the synthesis of antibacterial materials. At present, there are various nanoformulations composed of CuNPs under research to study applications in pulmonary infections.

Iron-based nanoparticles have found wide applications in the antibacterial field. The antibacterial mechanisms of iron ions are mainly as follows. Iron ions pass through the bacterial membrane through active or passive absorption, and then Fe^2+^ generates highly reactive hydroxyl radicals through the Fenton reaction. The free radicals not only lead to LPO production and GSH depletion, but also cause damage to DNA, proteins, and lipids, and finally lead to bacterial death (Gudkov et al. 2021). This ferroptosis-like antibacterial therapy has become an emerging effective antibacterial approach. Besides, iron nanoparticles have the unique physical properties of super paramagnetism. They are endowed to absorb magnetic field energy and convert it into thermal energy, thus killing bacteria as the temperature increases (Wang et al. 2023). The ferroptosis-like antibacterial therapy was verified in acute MRSA pneumonia (Shen et al. 2020; Hu et al. 2023), which sparked research interest for its applications in pulmonary infections.

Metal-based nanomaterials have enormous potential for antibacterial capability and pulmonary delivery, given that they have excellent and flexible properties and potential antibacterial activity. Sawatdee et al. (Changsan et al. 2024) prepared Col-CS-AuNPs from gold nanoparticles terminated with colistin and chitosan and encapsulated them into the MDIs to study their performance against pulmonary infections. Gold nanoparticles can kill bacteria and also potentially mitigate colistin toxicity, and Col-CS-AuNPs exhibit suitable aerosol properties for pulmonary drug delivery when developed as MDIs. Moreover, there would be other mental materials which can be utilized in the antibacterial field.

However, the safety of metal-based nanoparticles remains further investigation. The study on the biodistribution and clearance of inhalable metal-based nanomaterials are still not very sufficient and clear. Recently, a few investigations focused on the biodistribution following pulmonary exposure to silver, gold, copper oxide. It demonstrated that silver and gold were retained in the lung for an extended period (for 83.3 days and 25 days for silver and gold, respectively), while copper returned to baseline at 20.8 days in animals (Hadrup et al. 2025). Chronic exposure limits the application of the mental. Long-term metal clearance is mainly mediated by macrophages, and long-term exposure to metal ions may induce the occurrence of inflammatory responses (Nayek et al. 2022). The same safety concerns also happen when repeated inhalation occurs. The toxicity of metal-based nanoparticles was reported to be dependent on many factors, including size, shape, surface and contact interactions. Exposure to these nanoparticles may be toxic to cells due to the accumulation of ROS. The production of ROS could cause oxidative stress, inflammation and consequent damage to proteins, cell membranes and DNA, resulting in cell toxicity (Sengul and Asmatulu 2020). Previous studies have shown that biological safety can be adjusted by reducing the dose of metal ions and surface modification (Ali et al. 2020). Developing responsive metal-based nanoparticles can achieve targeting and precise delivery, which could decrease the likelihood of harmful side effects on beneficial bacteria or human cells, and ultimately generate more effective antibacterial therapeutic effect (Zhong et al. 2022). Alternatively, metal-based nanomaterials and other nanomaterials can be combined to form nanoformulations, which improve their efficacy and biocompatibility while decreasing metal-based nanoparticles aggregation (Luo et al. 2024). Overall, metal-based nanomaterials will offer excellent prospects in pulmonary infections.

### 3.2. Lipid nanomaterials

Due to the biocompatibility and biodegradability of lipids, lipid nanomaterials have gained tremendous interest in pulmonary infections. The lipid nanomaterials can be categorized into liposomes, niosomes, SLNs, and NLCs, and the following part would introduce the main species in detail.

#### 3.2.1. Liposomes

Liposomes are vesicular structures with concentric bilayers, mainly composed of hydrophilic and hydrophobic layers, which can be used to encapsulate hydrophilic or hydrophobic drugs. Because the bilayer structure of liposomes is similar to cell membranes, liposomes have good biocompatibility and biodegradability (Li et al. 2023). Moreover, liposomes are easy to fuze with the bacterial cell membrane and can release the encapsulated drug into the cell membrane or inside the bacteria, which is conducive to the internalization of drugs (Scheeder et al. 2023). Functionalized modification of related groups on the surface of liposomes can achieve targeted delivery of drugs, limit their distribution to healthy tissues, and minimize potential side effects (Bassetti et al. 2020). Moreover, liposome-encapsulated antibiotics have been found to effectively overcome enzymatic degradation, efflux mechanisms, and impermeable outer membranes (Ghosh and De 2023). Above all, liposomes are excellent candidates in clinical applications due to their strong loading capacity, excellent delivery performance and good biocompatibility.

Especially, the outstanding biocompatibility endows liposomes with wide applications, which cast light on the pulmonary infections. Specifically, some substances endogenous, as components of the lung, with surfactant and other unique physicochemical and biopharmaceutical properties can be used to prepare liposomes. This means that superior tolerability of liposomes in the pulmonary airways can be guaranteed (Pilcer and Amighi 2010). The application of liposomes in pulmonary delivery has been investigated by numerous studies and some liposome-based pulmonary formulations that have reached various phases of clinical trials. However, the development of inhalable liposomes faces technical challenges, biological challenges, and formulation challenges. In detail, achieving adequate deep lung deposition and targeting the drug to specific pulmonary airways, is still very tough, the condition of disease severity, patient’s age, breathing pattern and device configuration should be taken into account in developing the formulation. What’s more, the drug leakage and stability issues during storage and administration (e.g., nebulization) should be noticed. A lot of factors also need to be paid attention to, some detail clarifications can refer to related reviews (Bassetti et al. 2020; Mehta et al. 2020).

#### 3.2.2. Solid lipid nanoparticles

Solid lipid nanoparticles (SLNs) have been studied as a viable alternative to liposomes for drug and gene delivery to the lungs. Compared with liposomes, SLNs are likely to embed hydrophobic drugs, and they can remain solid at 37 °C to ensure in vivo stability and have better physical stability before and after atomization (Costabile et al. 2024). However, the low drug loading and drug leakage during storage are the main disadvantages of SLNs (Subedi et al. 2009). As the development of technology, nanostructured lipid carriers (NLCs) were developed, which were considered as the "second generation" of SLNs. The NLCs consist of a liquid lipid matrix surrounded by a solid lipid shell and have higher encapsulation efficiency and better drug release performance (Weber et al. 2014). Like liposomes, SLNs and NLCs are also excellent candidates for pulmonary delivery, but the difference is that in most cases, formulations of SLNs and NLCs are mainly administered by nebulization, and some DPIs have also been developed (Wang et al. 2021).

### 3.3. Polymer nanomaterials

Polymers are multifunctional biological materials with good biocompatibility and can be used as excellent drug carriers. Polyethylene glycol, hyaluronic acid, chitosan, dendrimer and zwitterionic polymers can self-assemble into nanoparticles with different shapes and sizes. As excellent nanomaterials, polymers show great potential in the antibacterial field. The polymer materials can encapsulate various antibacterial drugs by the simple embedding strategy to form nanoparticles. Besides, some synthetic polymer materials could be endowed with intrinsic antimicrobial activity by mimicking the structure of AMPs, while some polymer materials were conjugated with conventional antibiotics.

Driven by the significant advancements in controlled polymerization techniques, there has been growing interest recently in developing antibacterial polymeric candidates against pulmonary infections. With the development of bacterial resistance, AMPs have been used as an emerging alternative therapy for antibiotics, and the polymer-AMPs platform can be a considerate approach to achieving the ideal antibacterial effect. The polyethylene glycol-drug conjugates, chitosan-drug conjugates, and hyaluronic acid-drug conjugates have received extensive attention (Marasini et al. 2017). Conjugates of chitosan derivatives with AMP-dendrimer (AMPD) were formed through thiol-maleimide reaction. AMPD-chitosan conjugates exert a synergistic effect against *PA* by disrupting the outer and internal Gram-negative bacterial membranes (Patrulea et al. 2022). Besides, the covalent bond between AMPs and biocompatible polymers can be designed with responsive properties, so that the connector site could respond to the microenvironment of infection and the nanoparticles could release the drug in the ideal site (Pola et al. 2023).

Nowadays, polymer nanoparticles have been investigated for the treatment of pulmonary infections. García et al. (Agarwal et al. 2018) prepared phage-loaded polymer nanoparticles, which were deposited throughout the lungs by DPIs. The active phages in the formulation effectively killed bacteria and reduced infection and associated inflammation in wild-type PA and cystic fibrosis transmembrane conductance regulator knockout mice. Pini et al. (Cresti et al. 2022) encapsulated the AMP SET-M33 in polymeric nanoparticles, and the formulations were aerosolized and delivered to the lungs. The encapsulated peptide showed long-lasting antimicrobial activity was much less toxic than the free one, and lasted for up to 72 hours in vivo. The development of drug-encapsulated polymeric nanoparticles for pulmonary delivery has achieved considerable antibacterial effects.

However, the safety of polymers on cells remains a major concern. Therefore, experiments in vivo are needed to test the safety of polymer nanoparticles on lung lineage cells. In addition, the immune response induced by polymer nanomaterials and the application scope in animal models still need to be further studied (Lam et al. 2018), as well as the biological distribution and metabolic problems. These challenges provide us with new directions for further in-depth research on the application range of antimicrobial polymer nanoparticles, which is expected to provide new solutions for the development of inhalable nanoformulations for pulmonary infections.

### 3.4. Organic-inorganic hybrid nanomaterials

#### 3.4.1. Mesoporous silica nanoparticles (MSNs)

MSNs are formed based on silica and have a stable skeleton, a high specific surface area, and a large pore volume, which can load ten-folds more drugs than non-porous silica nanoparticles (Dos Santos Ramos et al. 2018). Owning to the stability, hydrophilicity, biocompatibility and dispersion, MSNs are extremely ideal biomaterials for delivering antibacterial drugs (Wang et al. 2019).

In the field of drug delivery, MSNs have significant advantages and are very suitable for the delivery of antibacterial drugs, including, (i) controllable particle size (50-300 nm) with good cellular uptake; (ii) the large pore volume and internal surface area, thus making the MSNs good drug carriers; (iii) a robust matrix structure resistant to thermal, pH, hydrolysis-induced decomposition and mechanical stress; (iv) selective surface functionalization for targeted delivery and controlled release; (v) unique porous structure inhibiting the quick release of loading components when their pores are not fully covered (Kesse et al. 2019).

MSNs are considered promising candidates for next-generation nanomaterials due to their unique drug-delivery properties, particularly suitable for pulmonary delivery. The excess of silica exposure would induce the silicosis, the emergency of MSNs aimed to avoid the issue. MSNs are unstable in hydrolysis and gradually converted to silicic acid or polysilicon acid, which has good biological safety (García-Fernández et al. 2021). Amorphous silica is classified as a generally accepted safe material by the FDA. Moreover, MSNs are more biocompatible due to their porous structure, which reduces interactions with cells. Compared with amorphous silica nanoparticles, they degrade faster and therefore have lower toxicity (Narayan et al. 2018). In terms of drug delivery and biosafety, MSNs are expected to be good carriers for pulmonary delivery to antibacterial.

#### 3.4.2. Metal-organic frameworks (MOFs)

MOFs, also known as porous coordination polymers, are porous crystalline materials composed of organic ligands and metal ions/metal clusters linked by coordination bonds, with hydrophobic and hydrophilic porous surfaces that can flexibly respond to chemical and physical stimuli (Wang et al. 2018). Compared with other types of nanomaterials, including porous materials, MOF has significant advantages, such as ultra-high surface area, adjustable uniform porous structure, abundant multifunctional sites, and diverse surface modifications. MOFs, as carriers, show strong loading capacity to various biomolecules, like lipids, oligopeptides, nucleic acids, and proteins, which can be integrated into MOFs by linker conjugation, de novo encapsulation, passive diffusion, or surface adsorption (Zhuang et al. 2017). The unique properties of MOFs can meet the needs of biomedical development, making them an emerging nanomaterial.

As promising nanomaterial, MOFs meet the required requirements for the treatment of pulmonary infection. On one hand, MOFs can perform antibacterial effects via several mechanisms, including controlling the release of antibacterial metal ions, releasing the antibacterial active ligands or the encapsulated antibacterial active species. Many primitive MOFs can be directly used for antibacterial, such as ZIF-8 (Zhao et al. 2022), MIL-100 (Fe) (Karmakar et al. 2024), etc. What’s more, complexes formed by MOF and other materials, such as silver, gold, and GO nanostructures, have specific composition, structure, physical and chemical properties, and are capable of performing synergistic functions to enhance antibacterial properties (Yan et al. 2022).

On the other hand, MOFs have highly customizable properties (geometry, number of pores, pore size, etc.) and can be used as carriers for pulmonary drug delivery. MOF-based inhalation preparations mainly focus on the form of DPIs in that MOFs could achieve consistent drug loading and polydispersity DPIs required (Zheng et al. 2022). The most commonly used carrier is CD-MOF. The highly uniform and regular morphology of porous CD-MOF crystals can control the particle size and also has high drug loading efficiency and sustained release ability. In addition, CD-MOF has good biocompatibility, and the safety of organic linker cyclodextrin has been widely used as a vehicle for pulmonary drug delivery (Zhou et al. 2021). Li et al. (Hu et al. 2019) developed nanoporous CD-MOFs, modified them with cholesterol (CHO) and formed them as DPI carriers for budesonide (BUD). In the study, they found that modification of the CD-MOFs with CHO could improve the flowability and particle aerodynamic behavior of DPIs. Good biocompatibility was confirmed on A549 cells. Other MOFs carriers are also under deep investigation. Remuñán-López et al. (Fernández-Paz et al. 2021) microencapsulated MIL-100 nanoparticles into mannitol microspheres (MS) to form the DPIs. They loaded the system with isoniazid (INH) and evaluated both the viability and safety of the drug vehicle components. Results have shown the inhalable nanoformulations displayed suitable aerodynamic characteristics for pulmonary administration and non-toxicity. Therefore, the proposed micro-nanosystem is a good candidate for the pulmonary administration of anti-TB drugs.

Overall, due to their customizable properties and favorable pulmonary delivery performances, MOFs are promising candidates for the treatment of pulmonary infectious diseases. Currently, most studies mainly focus on cheap metals as metal nodes, and few types of metals are involved. In the future, it is necessary to expand the variety of metal and organic ligands constituting MOFs and develop multifunctional organic ligands that combine coordination, activation, ligation, fluorescence, and therapeutic functions. More importantly, the current studies on the in vivo toxicity evaluation of MOFs are not in-depth. The CD-MOFs show good cell viability and biocompatibility in A549 cells, as well as good tolerance in rats. However, the safety of other MOFs materials is mainly verified in several cells, the safety in other line cells is still inconclusive (Hu et al. 2019). Therefore, the development of a MOFs-based drug delivery system with low toxicity, biodegradability, and good therapeutic effect is a prospective research direction, but there is still a long way to go.

In summary, the nanoparticles mentioned above are expected to be used in pulmonary delivery and exert favorable clinical effects. The advantages and limitations of the nanoparticles system are concluded in Table 1. At present, only one inhalable nanoformulation has been on the market, Arikayce^®^, which is delivered via nebulizer for the treatment of Mycobacterium avium complex (MAC) lung diseases. Another inhalable nanoformulation Linhaliq^®^ has been developed and recently tested in two phase III (NCT01515007 and NCT02104245), which was liposome-based formulation encapsulated with ciprofloxacin (Haworth et al. 2019). However, the majority of nanoformulations are under the preclinical stage, and the biocompatibility and toxicity issues should be taken into account. Besides, there remain some barriers that need to be addressed in the delivery process.

**Table 1.** Advantages, limitations and applications of inhalable nanoparticle-based delivery system for pulmonary infections in the preclinical stage.

Nanoparticle-Based delivery system	Advantages	Limitations	Refs.
Metal-Based Nanomaterials	Controlling morphology and adjusting particle size Owning antibacterial activity	Biocompatibility Long-term toxicity by repeated inhalation	(Nayek et al. 2022; Zheng et al. 2022)
Lipid Nanomaterials Liposomes Solid lipid nanoparticles	Biocompatibility and biodegradability Surface modifiability	Drug leakage during storage and administration Stability issues during storage and administration	(Mehta et al. 2020; Ghosh and De 2023; Li et al. 2023)
Polymer Nanomaterials	Biocompatibility Multifunctionalization Drug conjugation feasibility	Immune response Biological distribution and metabolic issues	(Lam et al. 2018; Li et al. 2024)
Organic-Inorganic Hybrid Nanomaterials Mesoporous silica nanoparticles (MSNs) Metal-organic Frameworks (MOFs)	Adjustable uniform porous structure Powerful drug-loading capacity Diverse surface modifications	Biocompatibility Chemical stability	(Narayan et al. 2018; Wang et al. 2018; Hu et al. 2019; Kesse et al. 2019; Fernández-Paz et al. 2021)

## 4. The barriers nanoparticle-based delivery system faced

**Figure 2.**
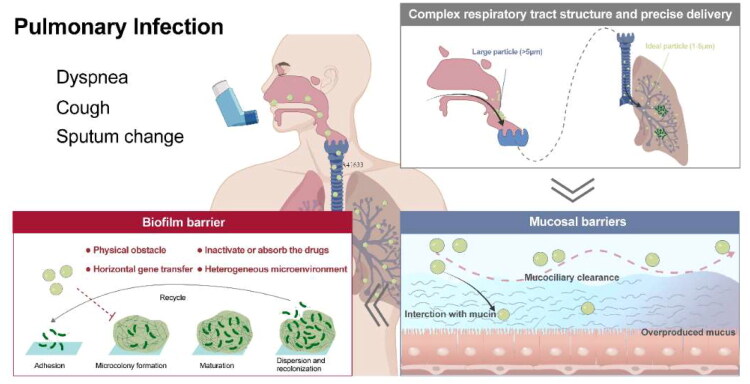
The barriers faced by inhalation in the treatment of pulmonary infection, including respiratory physiological structure, mucosal barrier and biofilm barrier.(originally created in adobe illustrator 2023).

Nowadays, inhalation is regarded as a critical treatment of lung infection-related diseases, which can make the drug better deposited in the lung lesions. However, the retention and deposition of drugs in the lungs face physiological and pathological barriers, and the complexity of lung structure makes the deposition of particles with different shapes and sizes in the respiratory tract different. When the drug remains at the lesion site, it may be cleared by coughing, mucociliary transport, and macrophage phagocytosis (Ruge et al. 2013), and then excreted from the respiratory tract. During the deposition in the lung, the drugs may suffer chemical, biological and physiological stress. In addition, bacteria will form biofilms, degrade inactivated drugs, promote autoimmune escape, and thus affect the effect of drugs. Therefore, we will discuss the barriers faced by inhalation in the treatment of pulmonary infections from three aspects: respiratory physiological structure, mucosal barrier, biofilm barrier and other barriers (Figure 2).

### 4.1. Complex respiratory tract structure and precise delivery

The human respiratory system includes several elements, including the corresponding part of the central nervous system, chest wall, pulmonary circulation, and respiratory tract (Person and Mintz 2006). In the famous Weibels model, the respiratory tract can be divided into 3 parts, including the tracheobronchial region, acinar region alveolar region and respiratory region, which can be further divided into 23 levels of complex and repeated bifurcated network structures. Another classification divides the respiratory tract into the upper respiratory tract and the lower respiratory tract. The bacteria-induced infection almost happens to the lower respiratory tract, which starts from the trachea and almost covers the entire lung. As the disease develop, the bacteria would colony deep in the lungs, and the infectious diseases become more severe.

Following the pulmonary administration, the particles would deposit in the pulmonary airways mainly via gravity sedimentation, inertial impaction, and diffusion (Liu et al. 2020). Each of these mechanisms is influenced by factors such as particle size, shape, and density, as well as airflow dynamics, and it determines whether the particles would deposit in the airway or exhale out (Kunde et al. 2022). Owning to the different anatomical structures of the lung, the fate of inhalable nanoformulations depends to a large extent on their deposition. The complex respiratory tract structure serves an important contribution to the precise deposition of the drugs.

Due to the bacteria colonies deep in the lungs, it is necessary to take measures to achieve effective drug deposition. However, if we only simply deliver abundant drugs to the lower respiratory tract, it may be the problem other administrations face. That is increasing concentration of drugs is always accompanied by increasing side effects. What’s more, it is likely to induce the development of drug resistance (Jin et al. 2023). To achieve a precious effect on bacteria is urgent, which could maximize the antibacterial effect and minimize the side effects.

Therefore, the complex respiratory structure makes it difficult to deposit drugs in the pulmonary, and the precise deposition of drugs in the lower respiratory tract is an urgent problem to be solved. After the drug is deposited in the target site, how to achieve the targeted-killing effect on bacteria is another key to exerting the ideal therapeutic effect.

### 4.2. Mucosal barrier

As the first line of defense against pathogenic infections, the mucus layer mainly plays a physical defense role, removing foreign substances through the interaction of mucus and cilia, which is named mucociliary clearance (MCC). The specific mechanisms are as follows: mucus captures particulate matter, including pathogens, and then the retained material is expelled from the airway through the ciliary bundles on the epithelial surface of the airway with the aid of rhythmic pulsation or coughing (Houtmeyers et al. 1999).

The mucus not only maintains the barrier function of the epithelium, respiratory mucus regulates the immune response, presents molecules with inhibitory effects on pathogens, and regulates cell differentiation and proliferation. However, it also acts as a transport barrier for drug delivery systems. The mucus mainly consists of water and mucins, with a significant amount of lipids and, a small amount of DNA under the pathological conditions (Creeth 1978). The biomolecules in mucus are cross-linked by disulfide bonds and/or physically entangled to form a meshwork with pore sizes in the range of hundreds of nanometers. The structure only allows particles of a certain size to penetrate the complex network. Mucins comprise an important component of airway mucus and contribute to the barrier function of mucus, and the mucins could link to polypeptides through the O-glycosidic bonding to form proteoglycans (Brockhausen and Schachter 1996; Brockhausen et al. 2009). The presence of –COOH, and/or –SO_4_^2-^ on the mucin proteoglycans endows an overall negative charge to the mucin layer (Li et al. 2013). The negatively charged glycosylated regions of mucin facilitate the interactions of mucins with positively charged particulates, which hinder the penetration of the particles. The viscoelasticity is considered a significant property of mucus. The high level of sialic acid accompanied with sulfate content would result in a strong negatively charged surface, thus increasing the rigidity of the mucus. Besides, the ratio of mucins to water ratio, and variation in the protein, ionic, and lipid content is critical for the regulation of mucus viscoelasticity. The change in the viscoelasticity could affect the MCC function, thus further influencing the adhesion of the particles. What’s more, the hydrophilic and hydrophobic domains throughout the mucus mesh further describe the movement behavior of particles in the mucus layer. The hydrophilic molecules interact with the mucus through the hydrogen bond interactions. The van der Waals interactions serve as the main force for the interactions with the mucus, and the induced electric dipoles generated on the polar as well as the non-polar molecules (Prasher et al. 2022). Therefore, the particles, which could interact with the mucins via electrostatic and hydrophobic forces, are not suitable for drug delivery due to the poor penetration toward the mucins.

In some respiratory diseases, such as asthma, COPD, and CF, the mucus is overproduced and its solid content augments, changing its structure and viscoelasticity (Abrami et al. 2024). For example, in the mucus of COPD patients, the mucin and other protein content highly increase, contributing to a higher mucus network and stronger interaction with particles. Moreover, the increasing viscoelasticity makes ineffective MCC as cilia cannot transport away the highly viscoelastic fluid, and the thick mucus becomes a static environment for the growth of pathogens (Chisholm et al. 2019). Besides, the mucus might trap the particles and block their access to the underlying epithelial surface. As a result, those changes in the mucus would cause damage to the airways and lead to a vicious cycle responsible for the harsh clinical evolution of these diseases. To sum up, to achieve the ideal delivery efficiency, the design of the nanoparticles should take the mucus transportation into account, mainly the particle size and the interaction with the mucins.

### 4.3. Biofilm barrier

Biofilm is formed by the accumulation of bacteria and their secretions that bacteria adapt to the natural environment, which act as a pathological factor. Bacterial biofilms are naturally resistant and impermeable to antibiotics due to their dense physical structure and polysaccharide- and nucleic acid-rich extracellular polymeric substance (EPS), thus increasing the difficulty of treating bacterial infection.

The mucus of the respiratory tract removes foreign pathogens through MCC and performs immunomodulatory functions. However, bacterial surface antigens can interact with mucins, thereby facilitating colonization, and nutrients in the mucins create a suitable environment for microbial growth. There is evidence that PA can secrete the virulence factor pyocyanin, which in turn increases the Sialyl - LewisX content on mucins, thereby facilitating PA-mucin interactions, making the host environment more favorable for attachment and colonization (Jeffries et al. 2016). Importantly, bacteria secrete extracellular matrix to defend themselves against the adverse external environment, which in turn encapsulates the bacterial cells in a self-generated polymeric matrix to form biofilm (Costerton et al. 1999). The biofilm provides a favorable environment for bacteria and aggravates the development of the disease.

The formation of biofilm is a dynamic process, which can be divided into four main stages: the adhesion, the microcolony formation, the maturation of biofilm, and the dispersion and recolonization. The specific process is as follows: 1) Adhesion: Bacteria use extracellular organelles such as flagella, cilia and mycelium and outer membrane proteins to adhere to or enhance adhesion to respiratory epithelium through extracellular polymers (Han 2013). 2) Microcolony formation: Under the regulation of the Quorum sensing (QS) system and cofactors, bacteria secrete various extracellular polymer components, including polysaccharides, DNA, lipids and proteins, and further form microcolony. 3) Maturation: As the bacteria proliferate, the biofilm gradually becomes mature. Mature biofilm has a diverse and unique metabolic structure that makes it resistant to harmful environmental factors and stress drivers. Mature biofilm has a spatiotemporal heterogeneous three-dimensional structure with a large number of pipes built around microcolony, which is interspersed in these structures to facilitate the exchange of nutrients and oxygen, the penetration of liquids, and the removal of toxins (Thorn et al. 2021). 4) Dispersion and recolonization: In the final stage of biofilm growth, some bacteria in the biofilm will actively disperse from the biofilm and search for new colonization sites. During this process, biofilm dispersion is affected by environmental signals, such as nutrients, nitric oxide, D-amino acids, Autoinducing Peptide (AIP), and Acyl Homoserine Lactones (AHL) (Rumbaugh and Sauer 2020). The dispersed bacteria return to the plankton mode, looking for new sites to restart the cycle. Therefore, the formation of biofilm is a cyclic process and biofilm makes the bacteria hard to be completely eradicated.

The formation of biofilm is closely related to the EPS by bacteria. The EPS predominantly comprises polysaccharides, proteins, extracellular DNA (eDNA) and lipids (Flemming and Wingender 2010). Together, they establish a cohesive, three-dimensional polymeric network that interconnects and transiently immobilizes the embedded bacteria (Flemming et al. 2023). Different components in the biofilm have different influences on the proliferation and diffusion of bacteria, while they have corresponding effects on drugs. Therefore, having a good understanding of the interactions could shed light on the methods to address the resistance. The following will introduce different components of biofilm.

Polysaccharides are the main components of the matrix, most of which are linear or branched structures of long molecules (Frølund et al. 1996), and many polysaccharides are found to attach to cell surfaces and form thin chains of complex networks that contribute to bacterial adhesion. Polysaccharides form the basic structure of biofilm and have diverse functions in the adhesion and microcolony formation stages. They can protect colonies within the biofilm from environmental stresses such as extreme environments, immune effects, or phagocytosis (Lahiri et al. 2022). At the same time, the complex network serves as a physical barrier, making it difficult for drugs to penetrate the biofilm to kill the bacteria.

The proteins in the matrix can be divided into enzymatic and non-enzymatic proteins. Many enzymes engaged in the degradation of polymers, they degraded the polysaccharides, proteins, nucleic acids, cellulose, chitin, lipids, and particles in the biofilm network structure (Flemming and Wingender 2010). Except for the degradation function, the enzymes can decompose the polymers into small molecular substances, which are converted into carbon and energy to feed bacteria in the biofilm. Moreover, the enzymes can adsorb or inactivate the drugs, resulting in preventing bacteria from being exposed to sufficient concentrations of the antibiotics. The above shows that enzymes play a positive role in the proliferation of bacteria. Non-enzymatic proteins in the matrix, such as cell surface-associated proteins and extracellular carbohydrate-binding proteins, are involved in the formation and stabilization of polysaccharide matrix networks and constitute the link between bacteria and EPS.

The matrix of biofilm contains eDNA, which is an important component of the matrix. eDNA is the main structural component in the biofilm matrix of *Staphylococcus aureus* and *Pseudomonas aeruginosa* (Yang et al. 2007) and plays an important role in the adhesion and primary stages of biofilm formation. The release of eDNA is regulated by the QS system. The normal generation of eDNA is closely related to the formation of biofilm and the release of virulence factors (Buzzo et al. 2021). Besides, eDNA plays a significant role in bacterial resistance. Negatively charged eDNA can interact with AMPs via electrostatically binding to inactivate them, and they can also inactivate antibiotics directly (Saxena et al. 2019). The inactivation effect would reduce the drug concentration in the biofilm, making the inefficient bactericidal effect.

Attributed to the interaction between the biofilm and the EPS, biofilm has multiple mechanisms that make it difficult to eradicate, resulting in severe resistance (Li et al. 2024). First, the biofilm serves as an obstacle, preventing bacteria from being exposed to sufficient concentrations of drugs. The complex spatial structure of EPS constitutes a physical barrier and impedes the diffusion of drugs. The polymers in the biofilm matrix bind with drugs and the extracellular enzymes can adsorb or inactivate the drugs. Besides, the biofilm has a heterogeneous microenvironment, including hypoxia and nutrient restriction, which makes the bacteria in the biofilm have lower metabolic activity or remain dormant. Since conventional antibiotics target the growth processes of bacteria, those inactive bacteria are not susceptible to antibacterial agents, showing high resistance (Flemming et al. 2016). Moreover, it has been hypothesized that there may be at least some bacteria in the biofilm with a specific, protected biofilm phenotype (Costerton et al. 1999). Furthermore, bacteria within the biofilm are liable to express specific genes to enhance bacteria tolerance (Ciofu et al. 2015). Due to the complexity of the biofilm, conventional antibiotics have limited ability to eliminate bacteria in the biofilm. To overcome the biofilm barrier, there is an urgent to develop new strategies to break through the biofilm matrix and control the release of antimicrobial drugs.

### 4.4. Other barriers

Other physiological barriers are also unneglectable, mainly including phagocytic of immune cells and the interactions with the related active components in the lungs. These barriers will affect the biological fate of inhalable formulations, thus influencing their therapeutic effects.

After the inhalable formulations deposite in the inhalable lower respiratory tract, they may face the phagocytosis and clearance from the macrophages and dendritic cells (DCs). This phagocytosis and clearance mainly depend on the size and surface properties of the particles. Specifically, particles with a size of 0.25–0.3 µm are possible for the uptake of macrophages, while the DCs tend to phagocytose the smaller particles (Geiser 2010). In addition, the immune cells also mediate the inflammatory responses by interacting with the particles. Therefore, the immunological barriers in the lower respiratory tract not only influence therapeutic effects of inhalable formulations but also could induce the safety worry (Wang et al. 2022).

In addition, the interactions between NPs and pulmonary surfactant (PS) are also important. PS is a surface-active material located on the thin aqueous layer lining the alveolar surface. PS is a lipid-protein system, and it mainly reduces the surface tension at the air-liquid interface to maintain the physiological respiratory function of the lung. Upon contact with the pulmonary surfactant, NPs may adsorb proteins and lipids in PS, thus forming biomolecular surfactant corona. These coronas significantly regulate the fate of inhalable NPs and the function of PS (Wang et al. 2022). Generally, NPs with smaller particle sizes, stronger hydrophobicity, and positive surface charge present higher lipid adsorption; hydrophobic NPs show a stronger interaction with SP-A; hydrophilic and negative NPs show a preferred interaction with SP-D (Liu et al. 2022). According to Hu et al. (Hu et al. 2013), the hydrophilic NPs generally translocate quickly across the pulmonary surfactant film, while a significant portion of hydrophobic nanoparticles is trapped by the surfactant film. The interaction will influence the process of interfacial transport of NPs and the delivery efficiency in lung. NPs transported rapidly across the PS film because of the weak interaction, while the hydrophobic NPs were trapped in the PS film due to the stronger interaction with PS. What’s more, the interaction between the PS and the NPs potentially affects the physiological function of PS. Once the key protein or lipid of PS is absorbed onto the NPs, it is necessary for maintaining the normal physiological respiratory. The damage or loss of these PS compositions will induce the function disorder of PS (Autilio and Pérez-Gil 2019). In conclusion, the fate of inhalable NPs and the function of PS were remarkably influenced by the PS-NPs interaction. Having a deeper understanding of the interaction may be conducive to increasing the delivery efficiency and exerting ideal therapeutic effects.

Proteases are known to play an important role in the normal function of the healthy lungs, as well as contributing to lung pathology in various disease states. The proteases play an extremely important role in the metabolic clearance after protein-based NPs inhalation. Compared with the health conditions, the expression of the proteases highly increased due to the immune response under the pulmonary infections condition. The proteases, such as neutrophil elastase (NE) and cathepsins, are released into the infection site, coupling with the enzymolysis action to the protein-based NPs (Woods et al. 2020). However, this degradation may not necessarily disrupt anti-microbial or immune modulatory functions of the protein. Creane et al. (2021) found that snake-derived AMPs were susceptible to cleavage by NE, while several retained their function following NE-incubation. Coupling with this found, they also verified that shorter AMPs are more resistant to degradation. Modifying with certain domains or encapsulated in inertia carriers were demonstrated the ability to prevent the active protein from degradation (Shoyele and Slowey 2006; Ngambenjawong et al. 2022).

Therefore, there are barriers faced by inhalation in the treatment of pulmonary infections: respiratory physiological structure, mucosal barrier, biofilm barrier and other barriers. We need to focus on the barriers and figure out corresponding approaches so that the effective antibacterial effects of the inhalable formulations would be achieved.

## 5. Effective strategies for overcoming the barriers

Inhalable formulations for the treatment of lower respiratory infections face multiple barriers to achieving effective antimicrobial effects. Combined with the delivery process, we should focus on how to achieve the effective concentration of drugs at the lesion site, which mainly concentrates on the following three parts: accurate deposition of lower respiratory tract, effective penetration of mucus and breaking of biofilm barrier.

### 5.1. Precise deposition in lower respiratory tract infection site

Due to the complex structure of the respiratory tract, the inhalable nanoformulations would encounter physiological barriers, which make it difficult to deposit in the targeted site. Indeed, the site and extent of aerosol deposition in the lung are dependent on particle size, velocity and inertia, the patient’s inspiratory air flow and the inhalation technique. With the development of technology, we can design certain formulations and devices to control the deposition of the inhalable nanoformulations. After achieving the deposition of inhalable nanoformulations in the lung, the realization of the target-killing effect on the bacteria is of equal importance. Next, we would introduce the related progress on the strategies.

#### 5.1.1. Control of the aerodynamic behavior of nanoformulations

With multiple deposition mechanisms, the inhalable particles with diameters within 1–5 μm are mainly deposited in the deep lung by inertial impaction and sedimentation. When the diameter is less than 1 μm, it may reach the alveoli by diffusion and sedimentation (Forest and Pourchez 2022). When the inhalable particles deposit in the lung, the loaded nanoparticles would then retain in the site. As many studies reported, the size range of nanoparticles should not be too large to ensure their deposition and then retention in the deep lung. Indeed, for pulmonary administration, the aerosol size of inhalable nanoformulations is the crucial factor that affects the deposition behaviors rather than the size of the nano-carrier systems. It is generally acknowledged that the optimal aerosol size for lung targeting is in the 1–5 µm range of aerodynamic diameters and lung flow rates should be 15–30 L/minute (Huang et al. 2021).

It has been reported that the aerosol size of the inhalable formulation is dependent on a wide variety of factors such as properties of the aerosol formulation and inhalation device features.

The influence of aerosol properties on lung deposition should be given great attention. As mentioned above, the main factor altering the deposition efficiency is the size of aerosol particles rather than that of nanoparticles. Particles with aerodynamic diameters ranging from 1 to 5 μm possessed excellent deposition efficiency. As well, the charge of aerosols plays an important role. For particles ranging from 0.1–1 μm, the net charge increases, coupling with enhanced deposition. However, for 2.5 μm particles, the net charge changes caused little difference on the deposition behavior, which might be due to the fact that large particles mainly deposited in the lung via inertial impaction (Koullapis et al. 2016; Bessler et al. 2023). Although much attention has been given to other parameters, particle shape effects have rarely been explored. Surprisingly, rod-shaped or fibrous particles such as asbestos and carbon nanotubes, are found the well deposition in the alveolar region compared with the circle particles (Mossman et al. 2011). The differences imply that particle shape enhances the aerodynamic behavior of particles. Several computational fluid dynamic (CFD) studies reported that fibers or elongated particles orient themselves parallel to the airflow, causing them to preferentially travel to the distal region of the lungs (Sturm and Hofmann 2006; Shukla et al. 2021). Deposition efficiency was also affected by the volatility of the particle. It seems to be true that condensation and evaporation behavior can change particle diameter and in turn, affect its deposition (Finlay 2021). Other factors, such as the properties of nanoparticles, would be elaborated in the following part.

Besides, the design of the device is more important. There are various advanced designs in inhalers, detailed information can refer to these reviews (Longest et al. 2019; Ari and Fink 2020; Abdelrahman et al. 2021; Komalla et al. 2023). The following is mainly focused on the relationship between devices choice and patients. The critical design modification function is to regulate the patient’s airflow within the device, thereby providing dispersion forces sufficient to overcome the inter-particulate force (Islam et al. 2024). The patient’s airflow also determines the selection of the devices. The pMDI and SMI typically advises the patient to breathe slowly and deeply and synchronize the device actuation with the inhalation, thereby increasing the retention time of formulation in the respiratory tract. Slow and deep breathes achieve a higher lung deposition efficiency of pMDI and SMI (Talaat et al. 2022). In contrast, DPIs, which rely on the patient’s airflow to initiate, require a faster and stronger airflow to optimally separate the drug powder from its carrier molecules. Faster and stronger airflow boosts the DPI’s deposition efficiency (Dhoble et al. 2024). Furthermore, respiratory rate, tidal volume and lung capacity can affect the retention time of aerosols in the lungs, and then affect their deposition rate. Respiratory rate, tidal volume and lung capacity can affect the retention time of aerosols in the lungs, then affect their deposition rate. Respiratory rate affects the residence time of aerosols in the lungs and, hence, the probability of deposition by gravitational and diffusional forces. The tidal volume alters the location where particles deposit in the respiratory tract (National Research Council Panel on Dosimetric Assumptions Affecting the Application of Radon Risk E 1991). The specific influences are as follows: 1) The total deposition amount decreases with the increase of respiratory rate. 2) Slow and deep breathing will evenly distribute aerosols in the lungs, but there is less aerosol deposition in the atmospheric tracts. 3) Rapid and shallow ventilation will enhance aerosol deposition in the atmospheric tracts and lead to the unevenness of the deposition distribution. 4) Slow and shallow breathing under high end-expiratory lung volume (EELV) can enhance the deposition of small airways (Heyder 2004). Jenny Rissler et Al. investigated the deposition efficiency related to breathing pattern and lung function in healthy children and adults. They present a linear regression model describing the deposition based on four variables: tidal volume, breathing rate, anatomical dead space and resistance of the respiratory system (Rissler et al. 2017).

#### 5.1.2. Precise target to the infection site

**Figure 3.**
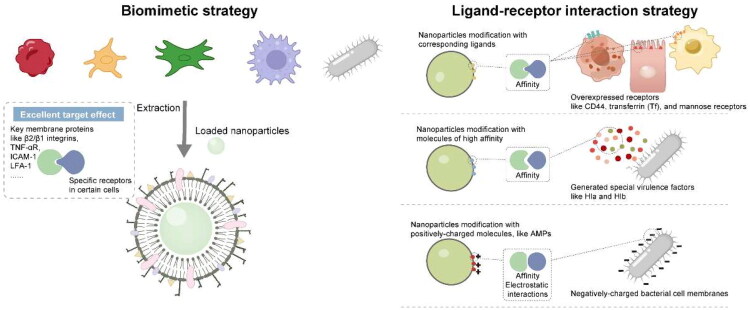
Schematic illustration of the precise targeting strategy to the infection site. (originally created in adobe illustrator 2023).

In the infection site, the foreign bacteria would damage the local cells and tissue and induce inflammatory responses at the lesion site. Then the inflammatory factors and chemokines released by local inflammation can recruit immune cells to the infected site. Inspired by the infecting process, we can find the following approaches to achieve the target delivery effect (Figure 3).

In recent years, the nanoparticle-biomimetic strategy has been investigated deeply attributed to biocompatibility. Due to the chemotaxis of immune cells, the bionic cell membrane-coated nanoparticles are regarded as excellent strategies for targeted delivery. The cell membranes of red blood cells, platelets, mesenchymal stem cells, neutrophils, macrophages, and bacterial-derived vesicles are deeply investigated (Zheng et al. 2022). Key membrane proteins such as *β*2/*β*1 integrins TNF-αR, ICAM-1, and LFA-1 on the surface of neutrophil cell membranes can bind to receptors on endothelial cells, which endow the nanoparticles of biomimetic neutrophil cell membranes the ability to specifically target inflammatory sites (Wang et al. 2020; Wu et al. 2022). Liu et al. (2023) constructed a neutrophilic bionic drug delivery system, which loaded gentamicin nanoparticles inside the cell membrane and coupled antibacterial peptides on the membrane, and it enhanced antibacterial activity against infectious pneumonia caused by drug-resistant Klebsiella pneumonia.

Another strategy is based on the ligand-receptor interaction. Certain sugars, peptides, proteins, or aptamers can bind to the specific receptor on certain cells, such as cancer, endothelial, or immune cells. Researches show that multiple receptors are overexpressed in lung cells when infection occurs, including CD44, transferrin (Tf), and mannose receptors (Wang et al. 2024). The summary of ligand-receptor targeting strategies to the infection sites is as follows (Table 2).

**Table 2.** The ligand-receptor targeting strategies to the infection sites.

Ligand	Targeting receptors	Application	Ref.
Sodium Hyaluronate	CD44 receptors	M.tuberculosis pulmonary infection	(Rossi et al. 2019)
Thioaptamers (TA)			(Leonard et al. 2017)
AFQ protein	Angiotensin-converting enzyme 2 (ACE2) receptors	SARS-CoV-2 infection	(Wang et al. 2024)
Ubiquitin-specific peptidase 2 (USP2)			(Dang et al. 2023)
α-hemolysin (Hla)	A disintegrin and metalloprotease 10 (ADAM-10)	USA300 infection	(Nygaard et al. 2012)
	Quinoxalinediones (QDS)	S.aureus infection	(Shekhar et al. 2025)
Plasminogen	Staphylokinase (Sak)	S.aureus pulmonary infection	(Kwiecinski et al. 2016)
Shed desialylated MUC1-ED	Flagellin	PA pulmonary infection	(Verceles et al. 2021)

In pulmonary infections, mannose receptors and CD44 are overexpressed on the surface of macrophages, and CD44 receptors can specifically bind to hyaluronic acid or chondroitin sulfate to achieve targeted effects (Valente et al. 2022). Godin et al. (Leonard et al. 2017) conjugated thioaptamers (TA) targeting CD44 to the mesoporous silicon microparticles’ surface to enhance the accumulation of particles in the infected site. Increasing 3-fold accumulations of CD44TA-SMP were recorded more than SMP group in *M. tuberculosis* for human macrophages, attributing to the affinity between CD44 and TA. In vivo, after CD44TA-SMP administration, *M. tuberculosis* CFU was reduced by 75% from the SMP control treatment. TA-targeted carriers significantly diminished bacterial load in the lungs and caused recruitment of lymphocytes. This CD44 receptor targeting strategy was successfully applied in pulmonary infections due to the overexpression of CD44 in macrophages under an inflammatory environment.

Human angiotensin-converting enzyme 2 (ACE2) membrane receptors were another attractive receptor under deep investigation, which were overexpressed in various cells of the human lung when infected by SARS-CoV-2 (Jia et al. 2021). Modifying the surface of nanoparticles with these ligands can target certain cells in the infection site. Xun Sun et al. (Wang et al. 2024) designed ACE2 receptor-targeted inhaled nanoemulsion (RDSV-NE-AYQ) to inhibit SARS-CoV-2. The optimized peptide AYQ had a superior binding ability to the ACE2 receptor. In vitro, NE-AYQ had a significantly higher uptake rate on hACE2 expressing cells and 24 times hACEs protein combination ability than the unmodified group. In vivo, NE-AYQ inhalation in hamsters had higher accumulations in lung tissues than unmodified group inhalation and remained in lung tissues for 72 h. This ACE2 receptor targeting strategy was successfully applied in other studies to treat the SARS-CoV-2 infection.

Besides, pathogenic bacteria can express their special virulence factors, which can be candidate targets for the nanoformulations. In *S. aureus*, α-Hemolysin (Hla) and *β*-Hemolysin (Hlb) are both important endotoxins, it has been reported that ADAM-10 (a disintegrin and metalloprotease 10) and sphingomyelin are cellular receptors for them, respectively (Jin et al. 2023). Plasminogen can be good ligands due to their affinity to staphylokinase (Sak) in *S. aureus* (Kwiecinski et al. 2016). There are different virulence factors in other bacteria, which determine the kind of target molecules. Therefore, conjugation or modification of these ligands to nanoparticles may be able to target the bacteria. In addition to the virulence factors target strategy, some positively charged materials have good affinity with the membrane of bacteria. Cationic metal materials, peptides and quaternary ammonium salts can bind to bacteria through electrostatic interactions, enhancing the concentration of antibiotic drugs and achieving targeted delivery (He et al. 2020; Fan et al. 2021; Jin et al. 2022).

There are many receptor-ligand targeting drugs on the market, which are mainly employed to treat tumors or autoimmune diseases, such as EGFR-targeted drugs gefitinib and erlotinib. However, based-ligand-receptor targeting drugs related to pulmonary infections are still in the preclinical stage.

In sum, the biomimetic strategy and ligand-receptor interaction strategy can efficiently target the immune cells or bacteria, thus achieving precise delivery to the infectious site.

### 5.2. Effective penetration of mucus

**Figure 4.**
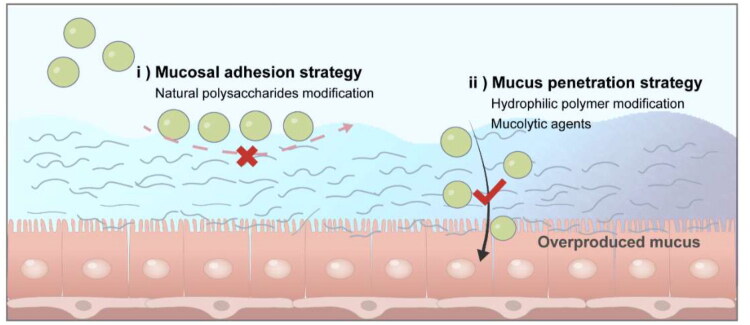
Schematic illustration of the approach to effective penetration of mucus, including the mucosal adhesion strategy and the mucus penetration strategy.(originally created in adobe illustrator 2023).

Due to the clearance of respiratory mucus, most drugs are cleared during the process of delivery, making the low-concentration drugs in the lungs. If the retention or penetration of drugs in the mucus layer can be effectively improved, it could achieve a higher targeted delivery to the respiratory tract (Murgia et al. 2018). The mucosal adhesion and mucus penetration strategies complement each other in the mucosal drug delivery system. Enhancing mucosal adhesion provides sufficient time for the drug to penetrate the mucus and finally reach the epithelial cell, thus increasing drug concentration in the target tissue and increasing concentration. Therefore, the design of nanoformulations with mucosal adhesion and penetration has a good application prospect in the treatment of respiratory disease (Dave et al. 2021; Watchorn et al. 2022; Yan and Sha 2023). The following part will emphasize the progress on the mucosal adhesion and mucus penetration strategies (Figure 4).

#### 5.2.1. Mucosal adhesion strategy

The mucosal adhesion process of drug carriers consists of two steps. First, the drug carriers close contact with the mucus layer, hydrate in the mucus layer, and retention in the mucus layer. Then the drug carriers diffuse or penetrate the mucus and enter the reticular structure of the mucus layer, ultimately interacting with the mucus layer in covalent or electrostatic adhesion mode. The carriers interact with the mucus via hydrogen bonds, disulfide bonds, electrostatic adsorption, hydrophobic force, or other van der Waals forces to achieve efficient mucosal adhesion. Whatever, the mucosal adhesion process is affected by various factors, including the molecular mobility, molecular weight, viscosity, and swelling characteristics of the mucus (Miyazaki et al. 2003; Jawadi et al. 2022). Facing many factors, exploring approaches to prolonging the whole process might increase drug dissolution and absorption.

Natural polysaccharides have high adhesion properties and can combine with mucins through intermolecular hydrogen bonding or van der Waals force. Natural polysaccharides, like chitosan, hyaluronic acid, and gelatin, are increasingly used in drug delivery, which was mainly attributed to their high biodegradability, bioavailability and mucosal adhesion properties (Yadav and Karthikeyan 2019; Balde et al. 2022; Valente et al. 2022). Recently, in order to improve the adhesion properties of nanoparticles, researchers have carried out functional modification strategies and proposed semi-synthetic adhesive materials such as chitosan derivatives and cellulose derivatives, etc. These materials mainly use carrier cationization strategies to enhance electrostatic attraction, such as the carriers by sulfhydryl or maleimide modification. The most widely used cellulose derivatives are hydroxyethyl cellulose, hydroxypropyl methyl cellulose and sodium carboxymethyl cellulose. As the research deepen, synthetic adhesive materials have been proposed, including carbomer, polyacrylic acid and polycarbophil (Khutoryanskiy 2011). Nowadays, chitosan derivatives and hyaluronic acid derivatives are widely used as excellent adhesive materials. The research progress of chitosan and its derivatives in mucosal adhesion is mainly introduced.

Chitosan is formed by deacetylation of chitin in an alkaline environment, and its primary amine is protonic and positively charged, which can interact with negatively charged mucins. When chitosan-based drug carrier reaches the mucus layer, chitosan could interact with the mucus via electrostatic force and ultimately retention in the mucus layer (Sogias et al. 2008). The chitosan is permeable in mucus, which hinders the particle’s retention in the mucus. Therefore, keeping the balance adhesion and permeability of particles modified with chitosan is the key point. And the permeability mainly depends on the degree of deacetylation and molecular weight of chitosan; there is a positive correlation between them. Besides, the interaction between chitosan and mucins also links with the adhesion. The polymer concentration, ionic strength, and pH value of the medium all play important roles. Take an example, the surface charge of chitosan will be screened in a high-salt environment, which is not conducive to binding with mucins (Mura et al. 2022).

To overcome these problems and further improve their mucosal adhesion properties, an effective strategy is to modify and derivate chitosan, such as thiolated chitosan, chitosan acrylate, trimethyl chitosan and carboxymethyl chitosan (M. Ways et al. 2018). Thiolated chitosan introduces thiol-containing ligands into chitosan, including thioglycolic acid, cysteine, NAC and GSH (Dünnhaupt et al. 2012; Mueller et al. 2012; Li et al. 2018), as the sulfhydryl group in thioglycolated chitosan forms disulfide bonds with cysteine residues of mucins, the adhesion between the carrier and the mucosa is enhanced. Kiss et al. (Kiss et al. 2022) synthesized chitosan-cysteine (Chit-cyst) conjugate and tested the adhesion by rheometer and texture analyzer. The adhesion between the Chit-cyst polymer and the mucin was greater than that of the chitosan powder.

#### 5.2.2. Mucus penetration strategy

Based on the barrier properties of airway mucus described previously, we can propose different strategies to enhance the penetration of drugs in the pulmonary mucus (Chen et al. 2021). Nanoparticles have significant advantages in improving cell penetration and are promising drug delivery systems for the treatment of lung diseases. As mentioned above, the mucus, as a barrier, hinders nanoparticle penetration, and the characteristic determines the penetrability of nanoparticles. Therefore, it is necessary to carry out appropriate designs for nanoparticles, such as changing the particle size and modifying them (including functional groups and charge density on the surface of the particles). In addition, disrupting the internal structure of the mucus can also increase the penetration of nanoparticles. The following will discuss the methods on how to increase the mucus penetration performance of nanoparticles.

##### 5.2.2.1. Hydrophilic polymer modification

Mucus has an amount of sialic acid and sulfate groups, making the mucus generally negatively charged. Thus, mucus penetration can be achieved by neutral or negatively charged hydrophilic polymer, which minimizes electrostatic and hydrophobic interactions (Taherali et al. 2018). Hydrophilic polymer modification endows nanoparticles with net neutral or negative charge, and it makes the weak interaction between the particles and mucins. Common polymers, like polyethylene glycol (PEG), polyvinyl alcohol (PVA), and carboxy-modified polystyrene and amphoteric polymers, can overcome the airway mucus barrier and deliver the drugs to the epithelial cell. Chai et al. (2020) reported the mucus-inert nanoparticles with the polystyrene (PS) and PEG to apply in pulmonary delivery. They prepared the PEG complex particles and transformed them to DPIs by spray drying. An 11-fold diffusion of PS-PEG nanoparticles was found in CF sputum than PS NPs. The single-particle tracks showed greater movement of PS-PEG nanoparticles, while PS NPs were confined to sputum.

**Figure 5.**
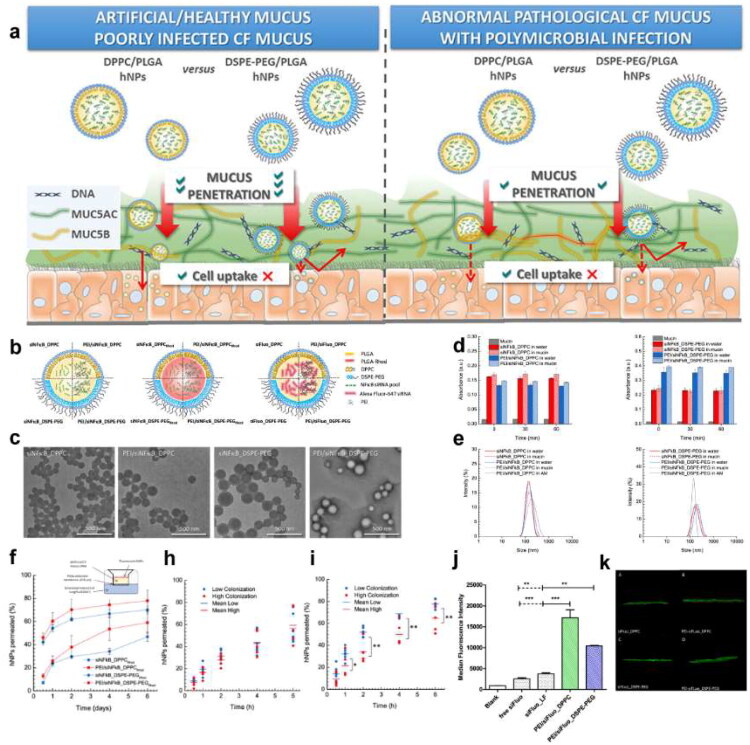
a) The mechanism of the hybrid lipid/polymer nanoparticles to tackle the mucus barrier; b) structure and composition of hNPs; c) transmission electron microscopy (TEM) image of the hNPs; d) scattering at 650 nm of hNPs (1 mg/mL) in the presence of mucin over time to assess the siNFkB-loaded hNPs interactions with mucin and artificial CF mucus(AM); e) size distribution by intensity of hNPs in the presence of mucin or AM evaluated by DLS; f) percent amount of siNFkB-loaded fluorescent hNPs permeated through AM in SILF over time; h–i) percent amount of PEI/siNFkB_DPPCRhod and PEI/siNFkB_DSPE-PEGRhod permeated through different CF sputum samples over time, respectively; j) cellular uptake in Calu-3 cells after transfection for 24 h with siFluo-loaded hNPs. As positive control, cells were transfected with siRNA/lipofectamine complexes (siFluo_LF). Free siRNA (free siFluo) was used as negative control; k) 3D view created from confocal laser scanning microscopy sections of confluent Calu-3 monolayers grown at ALI exposed to siFluo_DPPC(a), PEI-siFluo_DPPC(B), siFluo_DSPE-PEG(C), or PEI-siFluo_DSPE-PEG(D) hNPs for 24 h and subsequent staining of mucus with AlexaFluor488-labeled wheat germ agglutinin. Reproduced from Ref (Heyder 2004) with permission. Copyright 2022, American Chemical Society.

At present, PEG modification is widely used, but PEG can cause an immune response and hinder cell uptake, so it is necessary to find alternative substances instead of PEG (Chen et al. 2021). In recent years, the application of other polymers has shown broad application prospects. Conte et al. (2022) demonstrated that lipid-polymer hybrid nanoparticles (hNPs), including polylactic-glycolic acid (PLGA) and dipalmitoyl phosphatidylcholine (DPPC) lipid shells, can help nucleic acid transport through mucus. They reported that non-PEGylated (DPPC) nanoparticles can improve mucus penetration than PEGylated NPs. They utilized the artificial mucus and Calu-3 cell to test the mucus penetration performance, it was confirmed that non-PEGylated (DPPC) hNPs effectively diffused to the mucous layer and internalized by Calu-3 cell. These indicated that non-PEGylated polymers have good prospects for overcoming mucus and cellular lung barriers (Figure 5).

Therefore, it has been confirmed that hydrophilic polymer modification has promising applications in enhancing the penetration of nanoparticles. Some polymers, like PEG, garnered significant attention, whilst their side effects also need to be addressed.

##### 5.2.2.2. Mucolytic agents

Mucolytic agents mainly alter mucus rheology by disrupting the structure of mucins. In detail, related enzymes could cleave the structure of the mucins network to alter the intrinsic properties of mucus, thereby promoting drug penetration. According to the action mechanism, mucolytic agents can be mainly divided into two types: proteolytic enzymes and disulfide bond-splitting agents (Menzel and Bernkop-Schnürch 2018).

Proteolytic enzymes can hydrolyze the proteins in mucus, thereby reducing the viscosity of mucus. Common proteolytic enzymes include ambroxol, bromohexine (Deretic and Timmins 2019), chymotrypsin (Appel 1986), pepsin (Cai et al. 2021) and papain. Müller et al. prepared the nanoparticles based on papain-grafted polyacrylic acid (papain-g-PAA). The presence of papain on the surface and inside the particles strongly decreases the viscosity of the mucus, leading to facilitated particle transition across the mucus layer, while the permeation studies revealed that enzyme-grafted particles diffuse through the mucus layer to a 3-fold higher extent than the same particles without enzyme (Müller et al. 2014).

**Figure 6.**
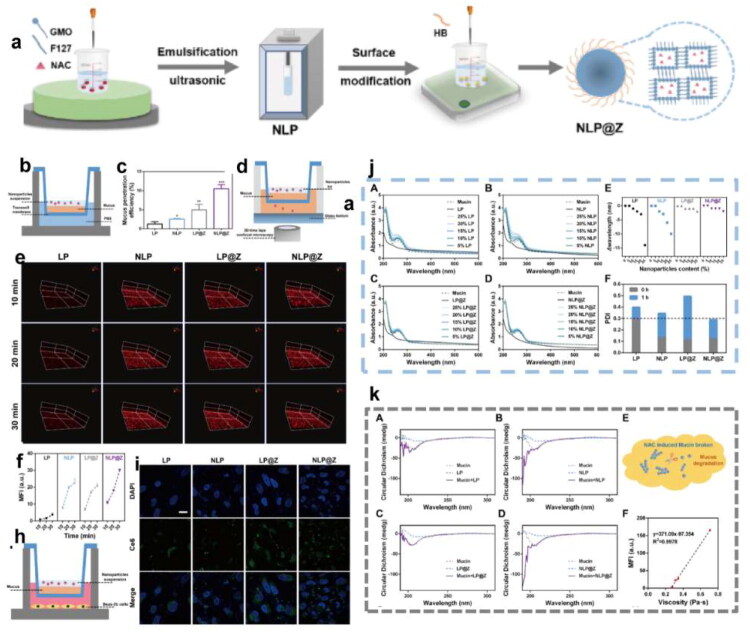
a) Schematical illustration of the preparation of NLP@Z; b) structure and composition of hNPs; c) the mucus penetration efficiency of different nanoparticles; d) D-time laps confocal microscopy for monitoring nanoparticle mucus penetration; e–f) the 3D-time laps confocal microscopy images of mucus penetration of different nanoparticles at different time intervals and its semi-quantitative results, respectively; h) schematic illustration of cellular uptake assay of mucus-penetrated nanoparticles; i) the CLSM images of cellular uptake of mucus-penetrated nanoparticles; j) the effect of mucus-inert surface of NLP@Z. A–D the UV–vis spectra of mucins after incubation with LP(a), NLP(B), LP@Z(C), and NLP@Z(D) of different contents. The Δwavelength changes of mucins incubated with nanoparticles of different contents(E). PDI values of nanoparticles before and after incubation with mucins (F); k) Mucin degradation effect of NLP@Z. A–D the CD spectra of mucins after incubation with LP(a), NLP(B), LP@Z(C), and NLP@Z(D). Schematic illustration of mucin degradation-induced mucus viscosity decrease (E). The linear regression between FB fluorescence intensity and microenvironment viscosity (F). Reproduced from Ref (Jia et al. 2021) with permission. Copyright 2023, Springer Nature.

Disulfide bond-cracking agents contain abundant sulfhydryl groups, and they split and cleave disulfide bridges between mucins through a chemical process of thiol exchange. Besides, they have a certain cracking effect on deoxyribonucleic acid fibers. By the mechanisms above, disulfide bond-cracking agents can reduce the viscosity of mucus and facilitate drug penetration. N-acetylcysteine (NAC) (Frye and Berk 2019) and carboxymethylsteine are the common disulfide bond-cracking agents. NAC, as a mucolytic agent, has significant efficacy in overcoming the airway mucus barrier. Kraegeloh et al. (Meziu et al. 2023) studied the effect of NAC on the mucosal penetration of nanoparticles. They cultured Calu-3 cells for 21 days to construct the mucus model, after that nebulized NAC was added to the mucus. Finally, they used the microscope to analyze the penetration of particles into the mucus. They found the cytotoxicity of particles increased after NAC treatment with an increased aggregation of nanoparticles in the mucus, suggesting that NAC compromises the integrity of the mucus barrier and its protective function. The results showed that the addition of NAC can increase the permeability of nanoparticles in mucus, so the functionalization of NAC on the surface of nanoparticles is an important strategy to enhance the efficiency (Nordgård and Draget 2018). Wang et al. (2023) proposed a lipid liquid crystal nanoparticle NLP@Z modified by cetylbetaine (HB) as a surface zwitterionic material and encapsulated by n-acetylcysteine (NAC). HB modification imparts a viscous inert surface to NLP@Z and inhibits the interaction between NLP@Z and mucin. NAC can effectively degrade mucin and further reduce mucus viscosity. This combined strategy has been shown to significantly promote mucus penetration performance and enhance epithelial cell uptake. In addition, the proposed NLP@Z has the required nebulization characteristics and can be used as a potential lung delivery nanoplatforms (Figure 6).

NAC was approved as an inhaled therapy for patients with CF and chronic bronchitis in the 1960s, due to the strong mucolytic property. However, the off-target irritation effects, including cough and bronchospasm, limit the application in clinical pulmonary medicine as an inhalable mucolytic. Therefore, it is advisable to find alternatives to overcome the limitation. An improved mucolytic P3001, P3001 acted faster and at lower concentrations than NAC, and it was more effective in CF sputum ex vivo (Ehre et al. 2019). Besides, a novel thiol-saccharide mucolytic MUC-031 has a more potent mucolytic, and it has little adverse effect compared with NAC due to the biocompatibility of carbohydrates (Addante et al. 2023).

Many studies were devoted to enhancing the effect of mucolytic, and properties of novel mucolytic molecules should include: 1) high efficiency/rates of enzymolysis, 2) prolonged residence times on airway surfaces (Ehre et al. 2019). In the future, we should aim to develop materials with excellent mucolytic properties to increase drug penetration in the mucus layer.

### 5.3. Overcoming the biofilm barrier

**Figure 7.**
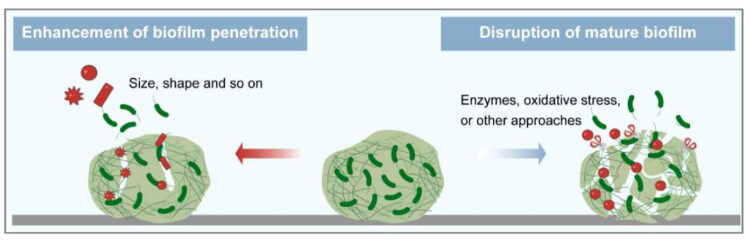
The strategy to overcome the biofilm barrier through the enhancement of the biofilm penetration and the disruption of mature biofilm. (originally created in adobe illustrator 2023).

Due to the structure and composition, the biofilm hinders the penetration efficiency of drugs and affects the therapeutic effect of antibiotics. Therefore, increasing the penetration of nanoformulations and reducing the interference of EPS become the keys to overcoming the biofilm barrier. Studies have shown that nanoparticles with specific sizes are conducive to the penetration of biofilm (Lv et al. 2023). Except for the penetration strategy, degradation of biofilm through physicochemical, oxidative stress, and other methods can also significantly enhance drug penetration (Figure 7) (Li et al. 2024).

#### 5.3.1. Enhancement of biofilm penetration

Size is critical for particles to penetrate the biofilm and the size of particles should not exceed the dimensions of the water channels in the biofilm matrix. It has been demonstrated that nanoparticles with a size of less than 350 nm can diffuse through pores within the biofilm, and the smaller the size of the nanoparticles, the stronger the penetration, the more readily cleared from the body. Therefore, the ideal size of nanoparticles ranges from 5 to 200 nm (Liu et al. 2019; Makabenta et al. 2021). For example, the EPS matrix can interact and prevent Ag^+^ from penetrating, but Ag NPs with a size of 10–40 nm can effectively penetrate biofilms, interact with pathogens and exhibit antimicrobial activity, showing brilliant eradication activity of *E. coli* and *Candida albicans* biofilms (Estevez et al. 2020).

**Figure 8.**
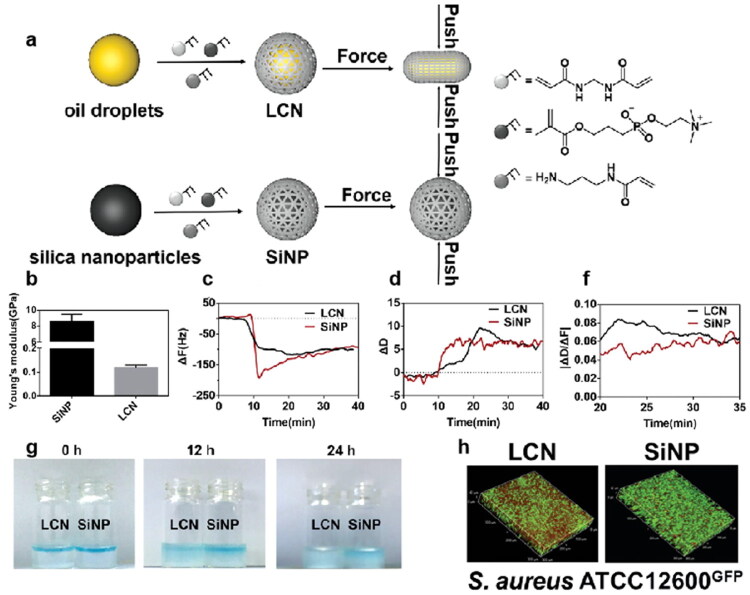
a) Schematic illustration of the preparation of LCN and SiNP, as well as the deformation caused by external forces; b) Young’s modulus of LCN and SiNP analyzed through AFM. c–f) ΔF, ΔD, and |ΔF/ΔD| of PBA-modified LCN and SiNP on galactose-coated Au sensor chip analyzed by QCM-D, respectively; g) The penetration behaviors of Cy5-labeled LCN and SiNP through 1% agarose gel; h) 3D confocal laser scanning of Staphylococcus aureus ATCC12600GFP biofilms with the addition of LCN or SiNP. Reproduced from Ref (Shekhar et al. 2025) with permission. Copyright 2023, Wiley.

Apart from the size of nanoparticles, the shape of nanoparticles also greatly affects their penetrating ability into the biofilm. For example, spherical nanoparticles may face less resistance from the EPS matrix than rod or linear nanoparticles, and exhibit higher permeability and dispersion uniformity (Chatterjee et al. 2021). Nanoparticles with unique shapes, such as nanoflower or sea urchin-shaped nanoparticles, can destroy the structure of EPS through mechanical forces (Linklater et al. 2021). Besides, shape-shifting nanoparticles can effectively penetrate biofilm barriers, which change into the corresponding shape according to the environment. Liquid core nanoparticles (LCNs), composed of polymer-coated edible oil droplets, exhibit high mechanical flexibility and can deform under external forces. The reports found that LCNs have a uniform distribution in the biofilm of green-fluorescent Staphylococcus aureus ATCC12600 GFP (Figure 8) (Wang et al. 2023).

In general, these strategies start from the properties of nanoparticles and have been applied to enhance the penetration of nanoparticles in biofilms and achieve better drug enrichment.

#### 5.3.2. Disruption of mature biofilm

Biofilm dispersion is considered a potential pathway for biofilm control. EPS formation is the basis of biofilm formation, which constitutes the scaffold of the three-dimensional structure of biofilm and provides a direct living environment for bacteria in the biofilm. Destruction of mature EPS can enhance the permeability of drugs in biofilm, thus improving the therapeutic effect of antibacterial agents. Therefore, the disintegration of EPS through enzymes, oxidative stress, or other approaches might be an effective way to overcome the biofilm barrier.

**Figure 9.**
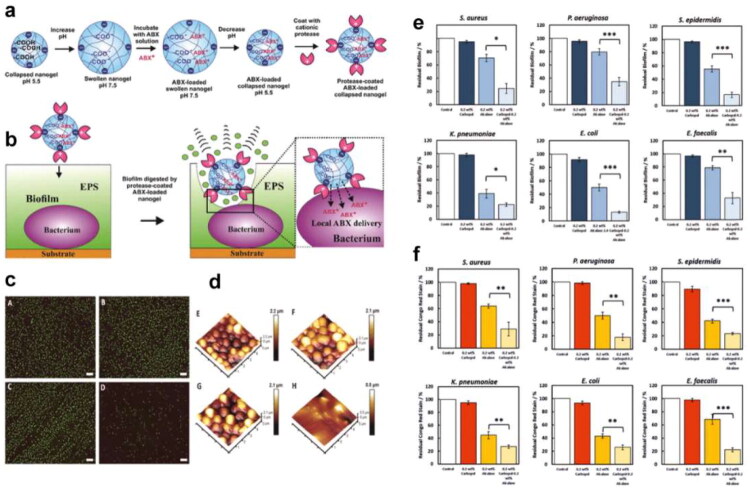
a) Schematic for the loading of the carbopol aqua SF1 nanogel with an antibiotic (ABX) followed by surface coating with protease (Alcalase 2.4 L FG); b) Diagram of the mechanism of action of the carbopol aqua SF1–Alcalase 2.4 L FG nanogel particles on biofilms adhered to a substrate; c) Fluorescent images of S. aureus biofilm formation, including untreated control sample (a), treatment with 0.6 wt % carbopol(B), 0.6 wt % Alcalase 2.4 L FG(C), and 0.6 wt % carbopol–0.6 wt % Alcalase 2.4 L FG nanogel particles(D); d) Tapping mode atomic force microscopy of 24 h growth S. aureus biofilms cultured on glass slides, biofilm growth control (E), treatment with 0.6 wt % carbopol(F), 0.6 wt % Alcalase 2.4 L FG(G), and0.6 wt % carbopol–0.6 wt % Alcalase 2.4 L FG nanogel particles(H); e) Ability of 0.2 wt % Alcalase 2.4 L FG and 0.2 wt % carbopol–0.2 wt % Alcalase 2.4 L FG to disrupt bacterial biofilms; f) Ability of 0.2 wt % Alcalase 2.4 L FG and 0.2 wt % carbopol aqua SF1–0.2 wt % Alcalase 2.4 L FG to disrupt the biofilm matrix protein by hydrolysis. Reproduced from Ref (Verceles et al. 2021) with permission. Copyright 2019, American Chemical Society.

EPS is mainly composed of proteins, polysaccharides and eDNA, which play a crucial role in maintaining the structure and function of biofilms. In a natural process, the bacteria in the biofilm can secrete extracellular enzymes, the enzymes would degrade EPS components, destroy the 3D structure of the biofilm and facilitate the bacteria to fall off the biofilm for further colonization (Rumbaugh and Sauer 2020). Inspired by this process, combining biofilm matrix-degrading enzymes with other antibacterial agents can increase drug concentration and elevate biofilm removal effectiveness. Paunov et al. (Weldrick et al. 2019) prepared protease-functionalized antibiotic nanogels, which were coated with protease Alcalase 2.4 L FG on the surface and encapsulated the ciprofloxacin inside. When the nanogels reached at the biofilm, protease would degrade proteins in EPS and destroy the integrity of the biofilm barrier, and then the diffusion of ciprofloxacin in biofilms was further enhanced. Proteases, including protease K, papain, trypsin and bromelain (Mugita et al. 2017; Pinto et al. 2020), can degrade the protein components in EPS and make the biofilm structure loose. Except for proteases, glycoside hydrolase and deoxyribonucleic acid also can destroy EPS matrix components. Glycoside hydrolases, such as mannosidase, cellulase, amylase and alginate lyase (Lv et al. 2023), can change the integrity of biofilm by degrading exopolycans. PelAh and PslGh can selectively target and degrade exopolycans in the biofilm matrix, which could destroy the original biofilm of PA within 1 hour, and inhibit biofilm formation within 24 hours (Figure 9) (Baker et al. 2016).

**Figure 10.**
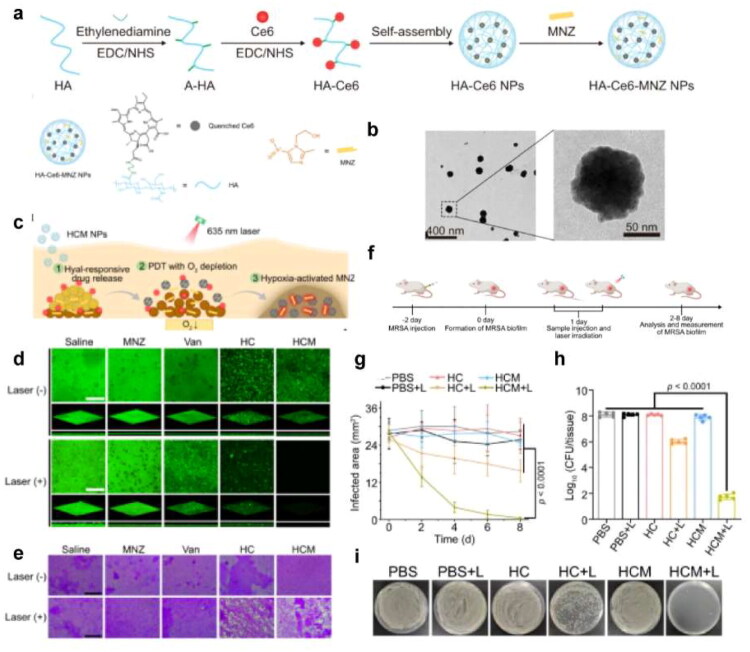
a) Preparation of HCM NPs; b) transmission electron microscopy (TEM) images of HCM NPs; c) schematic illustration of the PDT-induced hypoxia and subsequent activation of MNZ for enhanced anti-biofilm treatment by HCM NPs; d) 3D CLSM images of MRSA biofilms stained by Calcein-AM (green) after various treatments; e) micrographs of MRSA biofilms stained by crystal violet after various treatments; f) schematic illustration of the experimental procedure for treating MRSA biofilm infected mice; g) the infected area of the mice after various treatments; h) quantification of viable bacteria inside biofilm-infected tissues at 8 d post-treatment; i) photographs of MRSA colonies from infected tissues at 8 d post-treatment. Reproduced from Ref (Sogias et al. 2008) with permission. Copyright 2022, Springer Nature.

Oxidative stress against the biofilm includes reactive oxygen species (ROS) and reactive nitrogen species (RNS). Various advanced nanomaterials have been developed to generate abundant ROS and RNS in different ways, including nanozyme catalysis, photocatalysis, photodynamic therapy, sonodynamic therapy, and nitric oxide (NO) therapy (Liu et al. 2022; Li et al. 2024). ROS are the by-products of oxidative metabolism processes, including hydroxyl radical (·OH), hydrogen peroxide (H_2_O_2_), singlet oxygen (^1^O_2_), and superoxide radical anion (O_2_^–^). Materials such as semiconductor materials, nanozymes, photosensitizers and sonosensitizers can generate ROS under certain conditions. The produced ROS would damage biofilm and bacterial cells, and then nonselectively inactivate components (like eDNA, proteins and lipids), resulting in eDNA damage and protein inactivation (Dryden et al. 2017). Taking photodynamic therapy (PDT) as an example, the absorbed light energy exerts an antibacterial effect through two key mechanisms mediated by photosensitizers (PSs) (Songca and Adjei 2022). Specifically, the type I mechanism directly transfers hydrogen atoms to biomolecules through the free radical mechanism, and then the biomolecules react with O_2_ to generate ROS. In contrast, PSs in an excited triplet state can transfer energy to nearby oxygen to generate singlet oxygen in the type II mechanism. No matter what mechanism, it would cause irreversible chemical reactions that alter the function of biomolecules (Huang et al. 2012; Calixto et al. 2016). Wang et al. (Xiu et al. 2022) combined PDT and prodrug metronidazole (MnZ) to cure bacterial biofilm infection, they modified the HA with Chlorin e6 and MnZ and then prepared the HA-Ce6-MnZ nanoparticles (HCM NPs). In the *MRSA* infection model, under laser irradiation, Ce6 produced abundant ^1^O_2_, disassembled the biofilm, and exerted bacteria bacteria-killing effect in normal oxygen conditions. The PDT effect causes oxygen depletion and subsequently enhances hypoxia in the biofilm. Under hypoxia conditions, MnZ were activated and killed bacteria with low metabolic activity. This PDT-activated chemotherapy can be used to eradicate *MRSA* biofilm (Figure 10).

**Figure 11.**
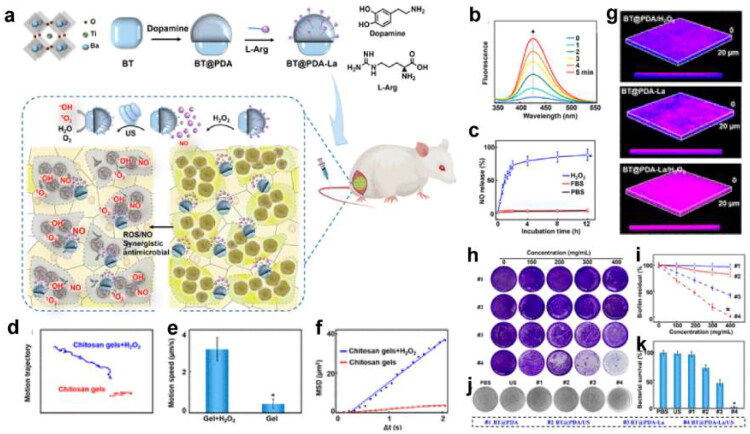
a) Preparation process and antimicrobial mechanism of BT@PDA-La NMs;b) •OH and 1O2 generation of BT@PDA-La NMs under US irradiation for 1–5 min; c) NO release of BT@PDA-La NMs in different media; d–f) motion behavior of BT@PDA-La NMs, including motion trajectories, speed, and MSD values versus time intervals of BT@PDA-La NMs in simulated biofilm matrix (chitosan gels) containing or without H2O2, respectively; g) integral CLSM images after layer-by-layer scanning after different treatments; h-k) photographs of crystal violet-stained MRSA biofilms (h), percent biofilm residual (i), typical colony units on the plates (j) and bacterial survival after different treatments (k). Reproduced from Ref (Taherali et al. 2018) with permission. Copyright 2024, American Chemical Society.

RNS is derived from the combination of NO and superoxide (O_2_^−^) radicals, which contain peroxynitrite (ONOO^−^), nitrogen dioxide (NO_2_), and dinitrogen trioxide (N_2_O_3_) (Szabó et al. 2007; Hu et al. 2020). RNS can aggravate the damage to bacteria by causing DNA cleavage and protein dysfunction through free radical peroxidation, which has stronger bactericidal activity than ROS (Deng et al. 2018). Nitric oxide therapy is regarded as an effective strategy for anti-biofilm. Nitrosothiols (RSNO), azodiol enoleates (NONOates) and nitrobenzenes (PhNO_2_) are common nitric oxide donor species, they can generate NO via different chemical reactions (Xiang et al. 2017). As a signaling molecule, NO regulates various biological processes and ultimately promotes biofilm dispersion. Besides, NO will further generate RNS and damage bioactive substances in the EPS matrix, thus exerting antibacterial and anti-biofilm effects (Schairer et al. 2012; Poh and Rice 2022). The ability of different NO donors to against the biofilm was investigated, and it was found that a certain threshold level of NO was required to achieve the ideal penetration effect. An appropriate level of NO would reduce the amount of biofilm generation, disperse the mature biofilm and change its mechanical strength (Grayton et al. 2023). Zhao et al. (2024) prepared a mushroomed Janus nanomotor (BT@PDA-La) driven by nitric oxide (NO). The nanomotor was coated with polydopamine (PDA) based on piezoelectric tetragonal barium titanate (BT), and the PDA was further modified with L-Arginine (L-Arg). In the infection microenvironment, L-Arg, as the NO donor, generated adequate NO to promote the nanomotor movement and promote its penetration in the biofilm. Under ultrasonic vibration, BT acts as a soundsensitor to elevate ROS level. The cooperative treatment of ROS/NO therapy destructed the biofilm and eradicated drug-resistant bacteria in vivo. Enzymes and oxidative stress can destruct the integrity of EPS, and make it possible for the nanoparticles to penetrate the biofilm into the infection site, thereby increasing the therapeutic effect of antibacterial agents (Figure 11).

In conclusion, the physicochemical methods and the oxidative stress approach can effectively degrade the biofilm and significantly enhance drug penetration. Currently, due to the rapid development of bacterial resistance, it highlighted that multipronged approaches might have potential to eradicate biofilms.

## 6. Summary and perspective

The treatment of pulmonary infections caused by bacteria is a great challenge, which imposes a huge economic and social burden on healthcare worldwide. In recent years, pulmonary drug delivery systems have effectively achieved the effective enrichment of drugs in lung lesions, which has been proven to have a good application prospect in the treatment of pulmonary infections. As drug delivery carriers, nanomaterials have shown significant advantages, such as increasing the delivery dose, achieving lung targeting and improving drug internalization, which can effectively realize the precise delivery of drugs and promote the further antibacterial effect of drugs. Inhalable drug delivery for the treatment of lung infections faces delivery barriers and the vulnerability of bacteria to drug resistance. Therefore, this article first reviews the physiological barriers faced by the infected lung and the employed drugs and then summarizes the strategies for overcoming barriers and emerging therapies, including increasing drug deposition in the lower respiratory tract, overcoming mucus and biofilm barriers, and emerging alternative therapies. Finally, combining the delivery and therapeutic aspects, we summarize the research progress of relevant nanoparticles for increasing drug delivery and achieving significant antimicrobial effects in pulmonary infections.

Despite extensive research on inhalable antibacterial nanoformulations for the treatment of pulmonary infections, there are still deficiencies. Much work needs to be done to facilitate clinical or industrial translation of inhalable antimicrobial nanoformulations. Some important issues require to be discussed in the clinical translation. First and foremost, the safety of the nanoformulations needs to be scrutinized. Different excipients can be formulated to endow the nanoparticles with certain characteristics to promote the stability of the formulation or improve the pulmonary delivery efficiency. At present, the FDA has approved some excipients, like chitosan and carboxymethylcellulose sodium, to apply in the inhalable formulation. However, many of the discussed excipients have not undergone rigorous toxicity testing, and further work must focus on holistic development and testing on novel excipients of inhaled dosage forms (Yousry et al. 2024). When it comes to the industrial translation, it still faces some obstacles. The preparation of the nanoparticles mostly is on the laboratory scale, which may be not consistent with the results on the industrial production scale. Further investigation should focus on the production process and ensure that industrialization can be achieved.

The majority nanoformulations are under the preclinical stage, assessments and predictions of the fate of the formulation in lung are increasingly essential. As several technical, methodological and analytical refine, in vitro and in vivo preclinical methods continue to evolve. As in vitro lung epithelial cell monolayer models, the air-liquid interface (ALI)-cultured Calu-3 cells have been used as a common cell barrier model to assess the transport of various drugs. Due to the ability to form highly-restricted monolayers, NCI-H441 and hAELVi cells have now been proposed as an excellent cell models (Sakagami 2020). As the in vitro lung tissue models, the primary ALI-cultured three-dimensional (3D) human lung cell barriers have also become available to simulate the mucus secretion and ciliary clearance functions, as well as the physiological barriers. The ex vivo isolated perfused rat lung (IPRL) methods have increasingly been used. Microfluidics holds potential for replicating the dynamics of human lung airway regions, like small airways and alveoli. Lung organs-on-chips have been extensively employed to study the delivery of inhaled substances within the lung’s complex microenvironment simulation. Besides, the breathing mucociliary-on-a-chip (BMC) platform effectively simulates the mucociliary clearance microenvironment and accurately replicates human respiratory dynamics in vitro (Lin et al. 2025). A multilayer lung chip with multigenerational alveolar ducts is available to investigate the inhalable particle deposition (Qiu et al. 2023). Nowadays, in vivo small rodent-based methods have been the mainstay use. Other animals are also utilized. Dogs’ respiratory frequency and tidal volume are close to human respiratory parameters, while sheep display several key features of human respiratory tract anatomy including the size of the nasal cavity and the airways. Non-human primates are considered as the gold standard model by the FDA for biopharmaceutical development (Sécher et al. 2020). In the global SARS-CoV-2 infection, rhesus macaque are used to assess the therapeutic effect of the inhalable formulations. However, primates necessitate specialized equipment and techniques along with ethical concerns. While there are various methods to simulate the real physiological conditions as much as possible, the consistency between the models and real human physiological conditions was still under investigated. In sum, there is still a long way to go to assess and predict the fate of the formulation in lung.

The future of inhalable nanoparticle-based delivery systems holds substantial promise with the integration of advanced technologies. Particularly, artificial intelligence (AI) provides continuous energy for the development of inhalable antibacterial formulations. Recently, the computer simulation technology has been extensively utilized in the drug screening. We can use the technology to integrate the data of the disease-related drugs in the large-scale database, construct models to analyze the correlation between the drugs and disease, and then screen out the active compounds with potential antibacterial agents. The technology would promote the development of new drug combinations and provide an efficient strategy for the treatment of pulmonary infections. Additionally, after having a preliminary grasp of the role of the contents in the formulation, machine learning could be utilized to analyze the correlation of every content in the formulation, which could deepen the understanding of the influence of each content and provide good guidance for the optimization of the formulations. Therefore, interdisciplinary research will drive innovation in creating novel formulations in the future. Furthermore, the essential element of the inhalable nanoparticle drug delivery system is the inhalation devices. The major factors that contribute to the delivery efficiency are the inhalation devices and the choice of inhalation device is very crucial because various factors could affect the process of delivery. And further technological improvements are needed to achieve precise delivery and meet the patient’s needs. What’s more, the stringency of evaluation should be improved, using clinically relevant animal models and clinical trials to evaluate. For example, the biofilm model mainly simulates biofilm formation, and there is still a certain gap with the actual biofilm. It is necessary to find a more relevant model for evaluation.

In summary, we should start from the pathological situation of pulmonary infections, combine the delivery strategy with alternative therapies, deeply integrate it with the nano-drug delivery system, and construct a three-step idea of "pathology-barrier-strategy", which will provide new insight for the research of inhalable antibacterial nanoformulations for the treatment of pulmonary infections.

## Data Availability

Details are available from authors.
